# Covalent fragment screening to inhibit the E3 ligase activity of bacterial NEL enzymes SspH1 and SspH2

**DOI:** 10.1039/d5cb00177c

**Published:** 2025-10-28

**Authors:** Cassandra R. Kennedy, Katherine A. McPhie, Aini Vuorinen, Jane Dudley-Fraser, Diego Esposito, Sarah Maslen, William J. McCarthy, Jonathan Pettinger, J. Mark Skehel, Jacob Bush, David House, Katrin Rittinger

**Affiliations:** a Molecular Structure of Cell Signalling Laboratory, The Francis Crick Institute 1 Midland Road London NW1 1AT UK katrin.rittinger@crick.ac.uk; b Proteomics Science Technology Platform, The Francis Crick Institute 1 Midland Road London NW1 1AT UK; c Crick-GSK Biomedical LinkLabs, GSK, Gunnels Wood Road, Stevenage Hertfordshire, SG1 2NY UK david.x.house@gsk.com

## Abstract

As the global fight against antimicrobial resistance in bacteria becomes increasingly pressing, new tool compounds are needed to study and evaluate novel therapeutic targets. Here, cysteine-directed fragment-based drug discovery is coupled with high throughput chemistry direct-to-biology screening to target the catalytic cysteine of a family of bacterial effector proteins, the novel E3 ligases (NELs) from *Salmonella* and *Shigella*. These effector E3 ligases are attractive as potential drug targets because they are delivered into host cells during infection, have no human homologues and disrupt host immune response to infection. We successfully identify hit compounds against the SspH subfamily of NELs from *Salmonella* and show that these proteins are inhibited by compound treatment, representing an exciting starting point for development into specific and potent tool compounds.

## Abstract 2

Identification of active site inhibitors of bacterial E3 ligases by covalent fragment screening coupled with high-throughput chemistry direct-to-biology (HTC-D2B).

## Graphical abstract

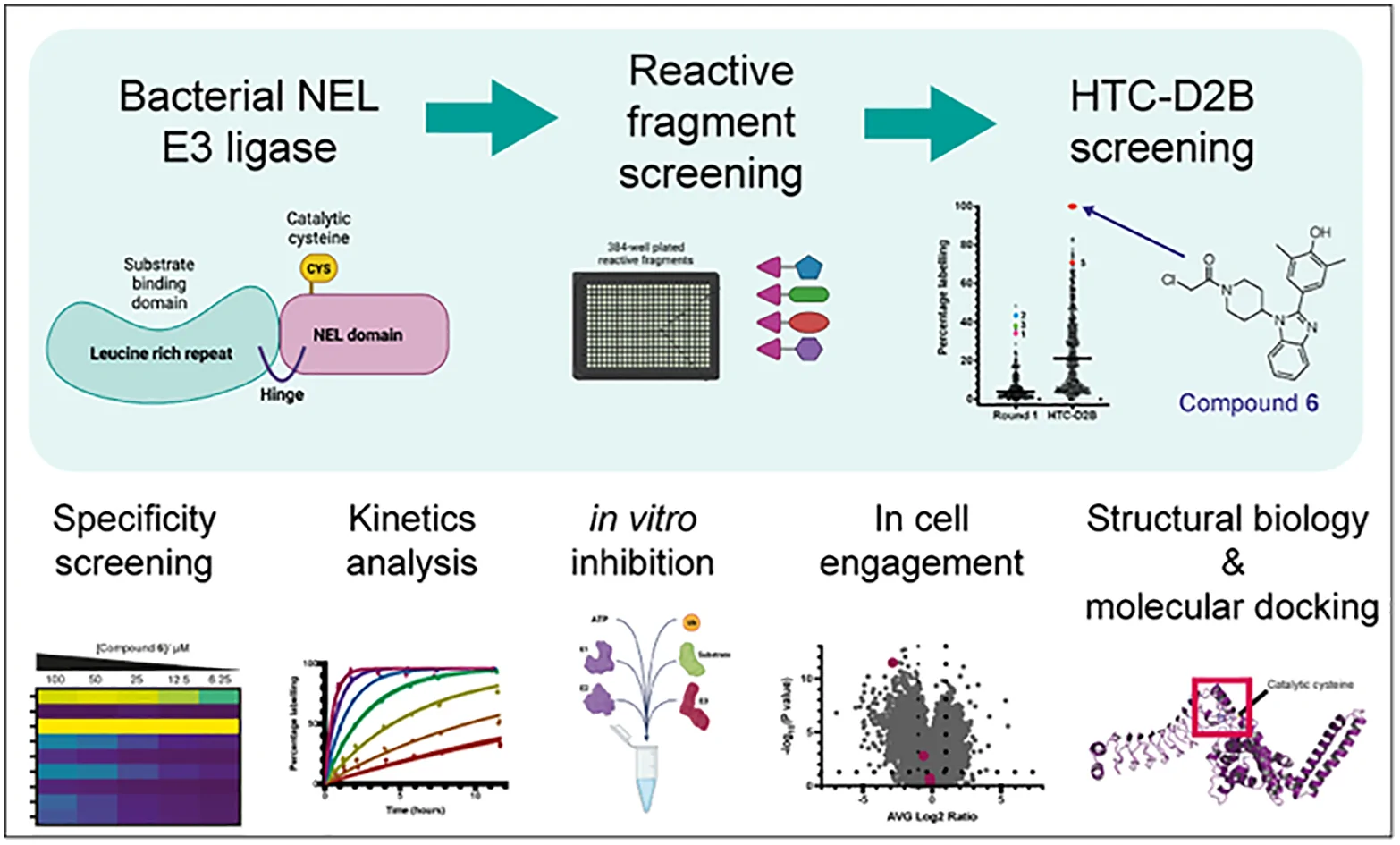

## Introduction

In eukaryotes, attachment of ubiquitin to lysine residues of proteins is an important mechanism to regulate cellular behaviour and signalling.^1^ Ubiquitin itself can be ubiquitinated, forming ubiquitin chains of different linear and branched topologies, that can elicit different cellular effects.^1^ An example of this is K48-linked ubiquitin chains, which mark the substrate protein for proteasomal degradation.^2^ Ubiquitin is added to substrate proteins *via* an enzymatic cascade of ubiquitin-activating (E1), ubiquitin-conjugating (E2) and ubiquitin ligase (E3) enzymes.^3–5^ E3 ligases determine substrate and ubiquitin chain specificity.^6^

Bacteria do not have their own ubiquitin system, however, many have evolved proteins that hijack the host ubiquitin system during infection.^7^ The novel E3 ligase (NEL) protein family comprises bacterial proteins which are delivered by some Gram-negative species through the type 3 secretion system (T3SS) into the host cytosol during infection.^8,9^ This family of proteins has been identified in *Salmonella* (SspH and SlrP proteins) and *Shigella* (IpaH proteins), as well as some less well-studied analogues in plant pathogens *Ensifer fredii* (NopM) and *Ralstonia solanacearum* (Rip proteins).^10^ These bacterial NEL E3 ligases subvert host E2 proteins and host ubiquitin to target host protein substrates that are important for the host defense system. Typically, these ubiquitinated host target proteins are then degraded by the host proteasome system thereby supporting bacterial survival and proliferation.^11–18^ By utilising the host degradation machinery, bacteria can disrupt the host immune response during infection with minimal energy expenditure.^19–22^

The NEL protein family has evolved separately to human E3 ligases, and therefore share no structural or sequence similarity in their catalytic domain to their host analogues. Mechanistic and structural studies have provided insights into this interesting family of proteins,^23–31^ however until now there have been no tool compounds or inhibitors available to study their activity *in situ*. Members of the NEL E3 ligase family share a highly conserved domain architecture, featuring an N-terminal LRR domain, which is responsible for substrate binding, a linker region, and a C-terminal NEL domain which contains the catalytic site and E2–Ub binding thumb.^24,25,27,28,31^ NEL proteins exhibit high interdomain flexibility, with different conformations observed in crystal structures and in solution.^23–31^ The NEL domain contains a catalytic cysteine which forms a thioester intermediate with ubiquitin before transfer to substrate lysine residues,^23^ analogously to eukaryotic HECT and RBR E3 ligases.^32,33^ In contrast to HECT and RBR E3 ligases, which often auto-ubiquitinate or form free polyubiquitin chains,^6^ bacterial NEL E3 ligases undergo non-productive ubiquitin turnover in the absence of substrate,^27^ which may deplete host reserves of activated ubiquitin during infection.

Whilst there has been a sustained recent focus on developing chemical probes against the entire human proteome, including initiatives such as TARGET 2035,^34,35^ there has been no corresponding campaigns for targeting bacterial proteins despite their capacity to impact human health during infection. Understanding and deciphering the role of bacterial proteins, such as NELs, and their tractability as therapeutic targets depends on the development of specific and potent tool compounds. With this in mind, we set out to find ligands of NEL proteins.

Fragment-based drug discovery (FBDD) is a powerful technique for tool compound development and drug discovery, that has been repeatedly and successfully utilised against eukaryotic ubiquitin system proteins.^36^ Despite this, FBDD is often limited by difficulties detecting weak target–fragment interactions, which is a result of small fragment sizes. One strategy to overcome this challenge is by deploying covalent FBDD, where an electrophilic warhead is appended to fragments. Covalent fragment warheads can be tuned for reactivity with different amino acid residues, and result in high occupancy covalent fragment–target interactions which can be robustly detected.^37,38^ We have previously used covalent FBDD to target HOIP^39^ and several deubiquitinases (DUBs),^40^ and since used HTC-D2B to rapidly advance our FBDD screening platform and increase screening throughput.^41–47^

We identified the NEL catalytic cysteine as a putative target for covalent tool compound development with a cysteine-directed covalent fragment-based screening campaign. Since bacterial E3 ligases are delivered into host cells during infection, any compounds targeting their activity would not need to cross the bacterial cell wall, making these proteins attractive drug targets. Herein we report the discovery and development of the first inhibitors of the bacterial NEL family of E3 ligases that show potent inhibition of *Salmonella* SspH1 and SspH2 proteins.

## Results and discussion

### Cysteine reactive fragment screening against IpaH9.8 and SspH1

**Fig. 1:**
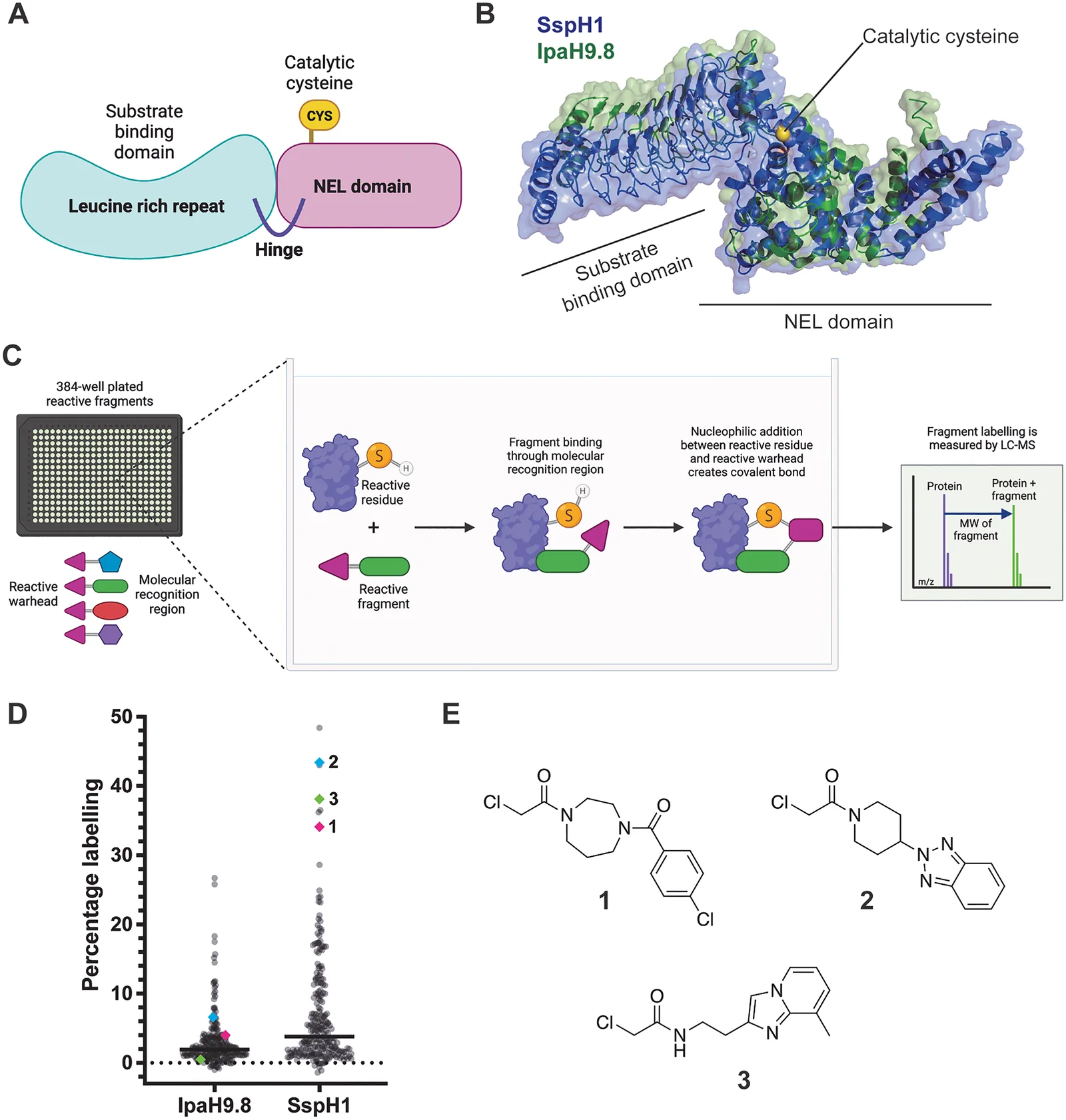
Intact protein LCMS screening of covalent fragment library against NEL proteins. (A) Cartoon depicting conserved NEL E3 ligase domains; (B) overlaid structures of SspH1 (blue, PDB 9H6W^30^) and IpaH9.8 (green, PDB 6LOL^29^) aligned on NEL domain; (C) workflow of covalent fragment screening using intact LC-MS; (D) fragment labelling percentages for SspH1 and IpaH9.8 with fragments 1–3 highlighted. Recombinant IpaH9.8 (21–545) and SspH1 (161–700) were incubated at 0.5 μM with 50 μM fragments for 24 hours at 4 °C, before fragment labelling was analysed by intact LC-MS; (E) chemical structures of fragments 1–3.

A diverse library of 227 compounds with chloroacetamide warheads, featuring a diversity of molecular weight (162–321 Da) and clog *P* (−1.4 to 3.4) (Table S1), were screened against *Salmonella* and *Shigella* NEL E3 ligases using our intact protein liquid chromatography mass spectrometry (LC-MS) platform as previously described.^39,40^ Briefly, recombinant SspH1 (161–700^30^) and IpaH9.8 (21–545^29^) (Fig. 1A and B) were incubated with 50 μM fragments for 24 hours at 4 °C before analysis by LC-MS (Fig. 1C). Raw counts were deconvoluted and the labelling percentage calculated by detection and comparison of protein and protein-fragment molecular weights.

Despite originating from different bacterial species, IpaH9.8 and SspH1 share an overall protein sequence similarity of 38%. The NEL domains alone have a 42% similarity, and so we expected to see common fragment hits between the two proteins. The IpaH9.8 construct contains three cysteine residues, while the SspH1 construct only contains two. We were surprised that IpaH9.8 was labelled significantly less than SspH1 (Fig. 1D); however, this likely results from inherent differences in activity and cysteine accessibility between the two species.^25,27,29,30^ Despite detecting no hits for *Shigella* IpaH9.8 with protein labelling greater than 30%, we identified several hits above this threshold for *Salmonella* SspH1 and therefore decided to focus further compound development on *Salmonella*. Of the 16 compounds that labelled SspH1 more than 30%, most were deprioritised due to multiple labelling events or because they were known promiscuous hits. However, three promising fragments (1, 2 and 3) were identified against SspH1 for further development (Fig. 1D and E, deconvoluted spectra in Fig. S1A and B).

### High throughput chemistry with direct-to-biology fragment elaboration for SspH1 targeting

To increase the potency *via* rapid elaboration of hit fragments for SspH1, we next sought to utilise a high throughput chemistry direct-to-biology (HTC-D2B) screening platform.^41,48–50^ Translating fragment hits into potent lead compounds traditionally relies on time-consuming medicinal chemistry campaigns. The HTC-D2B platform enables rapid synthesis and testing of compounds in a 384-well plate format, utilising a single step amide coupling reaction to convert amine building blocks into chloroacetamide functionalised fragments. Following reaction quenching, crude mixtures are screened directly against purified proteins, providing a high-speed alternative to individual synthesis and purification.

**Fig. 2:**
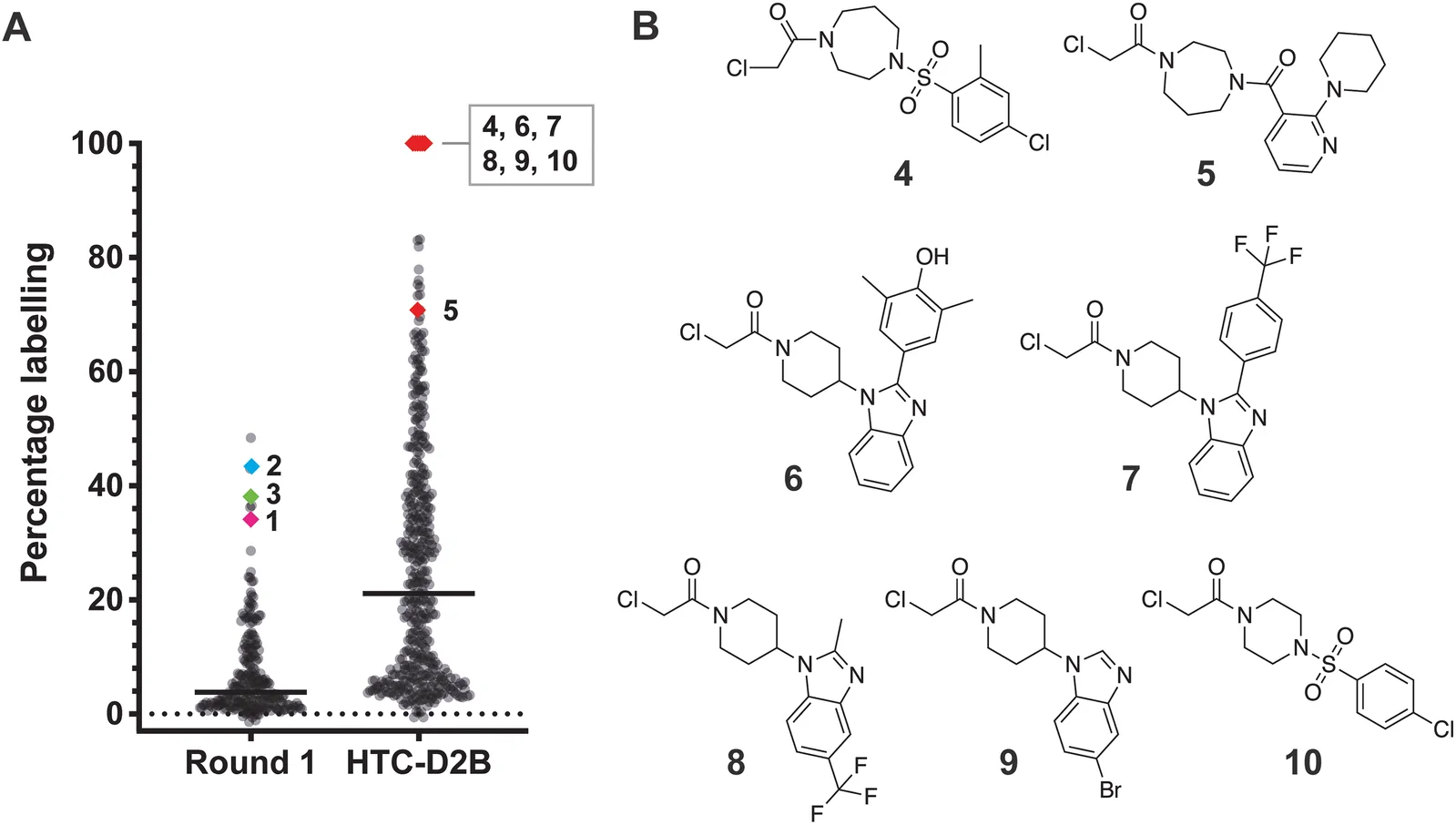
High throughput chemistry and direct to biology (HTC-D2B) optimisation for SspH1 hit fragments. (A) Labelling percentages for HTC compounds with compounds 4–10 highlighted in red compared to round 1 screening. Recombinant SspH1 (161–700) was incubated at 0.5 μM with 50 μM fragments for 24 hours at 4 °C, before fragment labelling was analysed by intact LC-MS; (B) chemical structures of compounds 4–10.

Amines related to fragments 1–3 were selected based on Tanimoto similarity constraints,^51^ and filtered for a molecular weight range of 130–350 Da. Anilines were removed to ensure compatibility with the HTC system. One library of 81 amines was designed based on fragment 1, and a second library of a further 349 amines based on fragments 2 and 3. The plated amines were then coupled with *N*-(chloroacetoxy)succinimide *in situ* at room temperature for 1 hour in a 384-well plate format to form their respective chloroacetamide reactive fragments. Extent of conversion was measured by LC-MS (Fig. S2). Following a reaction quench with hydroxylamine to remove unreacted succinimide ester, compounds were then directly incubated with SspH1 (without further purification) for direct-to-biology screening (0.5 μM protein, 50 μM fragments, 24 hours at 4 °C). Protein labelling was measured by intact MS as for the first round of screening. We were pleased to observe significantly improved labelling of SspH1 with second generation fragments, with six compounds exhibiting 100% labelling (Fig. 2A). From these improved hits, seven fragments (4–10) were selected for resynthesis and purification to enable further testing (Fig. 2B, deconvoluted spectra in Fig. S3).

### Hit compounds selectively label catalytic cysteine of SspH1 and SspH2

**Fig. 3:**
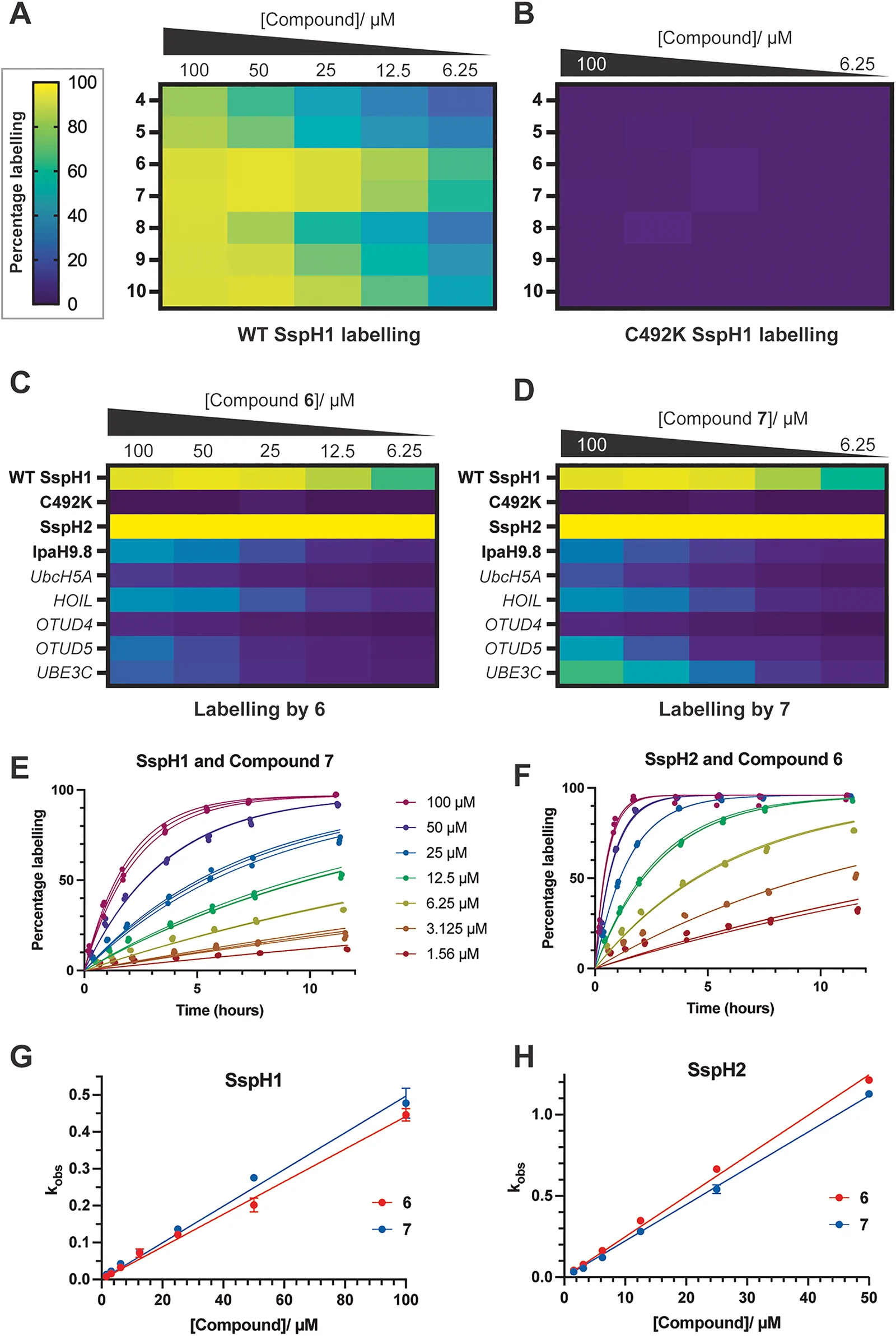
Validation of HTC hit compounds by intact MS. Labelling heatmaps of dilution series (100–6.25 μM) of compounds 4–10 with (A) WT SspH1 and (B) C492K SspH1. Labelling heatmaps of dilution series of (C) compound 6 and (D) compound 7 with bacterial and human ubiquitin binding proteins. Recombinant proteins were incubated at either 1.0 or 0.5 μM with 50 μM fragments for 24 hours at 4 °C, before fragment labelling was analysed by intact LC-MS. Bacterial proteins are shown in bold and human proteins in italic fonts. (E)–(H) Kinetics analyses: time courses (0–12 hours) of compound labelling (100–1.56 μM) of SspH1 and SspH2 (0.5 μM) for (E) SspH1 and compound 7, and (F) SspH2 and compound 6. Measurements were performed in technical triplicates. Labelling percentages were plotted against time in GraphPad Prism v.10, and curves fitted separately for each replicate using one-phase association, with constraints *Y*_0_ = 0 and plateau = highest labelling percentage. Graphs for SspH1 and compound 6, and SspH2 and compound 7 are shown in Fig. S4A and B. Rate constants (*k*_obs_, as given by Graphpad Prism calculated *K* values) were plotted against fragment concentration in triplicate for (G) SspH1 and (H) SspH2. Straight lines were fitted with constraint *Y*_intercept_ = 0. For SspH2 kinetics, 100 μM *k*_obs_ was outside the linear range. Data are presented as mean ± SD, *n* = 3. *k*_inact_/*K*_I_ values are reported in Fig. S4C.

We next analysed the potency of compounds 4–10 against SspH1. The seven compounds were obtained as purified compounds (Table S2) and screened against SspH1 by intact LC-MS (Fig. 3A) with two-fold serial dilution (100–6.25 μM). To confirm which of the two cysteines in the SspH1 161–700 construct were labelled, we repeated the concentration response experiment with a catalytic cysteine mutant of SspH1 (C492K) (Fig. 3B). The complete abrogation of labelling confirmed that all 6 compounds labelled the catalytic cysteine of SspH1. Compounds 6 and 7 were shown to be the most potent hits, with labelling of >60% at 6.25 μM and >80% at 12.5 μM (Fig. 3A).

To mitigate the risk of selecting compounds which indiscriminately recognise ubiquitin binding proteins, we next performed preliminary selectivity studies with compounds 6 and 7 against human ubiquitin system proteins. We incubated a dilution series (100–6.25 μM) of compounds 6 and 7 with human E2 UbcH5A, RBR-type E3 HOIL, HECT-type E3 UBE3C, and DUBs OTUD4 and OTUD5, and observed either minimal or no labelling by intact protein LC-MS (Fig. 3C and D). In parallel, we tested the compounds against related NEL E3 ligases SspH2 and IpaH9.8. We observed minimal labelling of *Shigella* NEL E3 ligase IpaH9.8 with either compound, but very strong labelling of *Salmonella* NEL E3 ligase SspH2. Our identification of dual SspH1 and SspH2 ligands is likely due to the high protein sequence similarity of SspH1 and SspH2 (60% for full length; 78% for NEL domains).

To further understand and compare the labelling of SspH1 and SspH2, we performed a full kinetic analysis of compounds 6 and 7 with both proteins (Fig. 3E–H and Fig. S4A, B). We observed a 4–5 fold higher *k*_inact_/*K*_I_ for both compounds with SspH2 than SspH1 (6.9 and 6.2 M^−1^ s^−1^ for SspH2 compared to 1.2 and 1.4 M^−1^ s^−1^ for SspH1), with little difference between the two compounds for either protein (Fig. S4C). Up to a maximum test concentration of 100 μM, the *k*_obs_*vs.* concentration plot was linear for SspH1 so it was not possible to derive individual values of *k*_inact_ and *K*_I_. For SspH2, the relationship was also linear up to 50 μM so it was still only possible to reliably calculate *k*_inact_/*K*_I_. Nonetheless, we can infer from these plots *K*_I_ values (SspH1) >100 μM and *K*_I_ values (SspH1) >50 μM for compounds 6 and 7. From these lower bounds on *K*_I_ it is clear that the range of ligand efficiencies reflecting the reversible binding interactions with SspH1 and 2 are low (0.19–0.21) and may hint at a challenging binding site for optimising a small molecule inhibitor. Identification of alternative hit series and/or X-ray crystallography would be expected to yield further insight into this putative small molecule binding site. To better characterise the intrinsic reactivity of these binders, we performed glutathione reactivity assays with both compounds, obtaining GSH *t*_1/2_ values of 4.9 hours for compound 6 and 4.0 hours for compound 7. Comparison of these to previously published GSH *t*_1/2_ values under the same conditions suggests that neither compound 6 or 7 has a high intrinsic reactivity (osimertinib *t*_1/2_ = 1.3 hours).^41^ At present we don’t understand the molecular basis for the increased reactivity of SspH2 over SspH1, and can only speculate that subtle differences in the active site environment render the catalytic cysteine more reactive.

### SspH1 inhibition with compounds 6 and 7*in vitro*

**Fig. 4:**
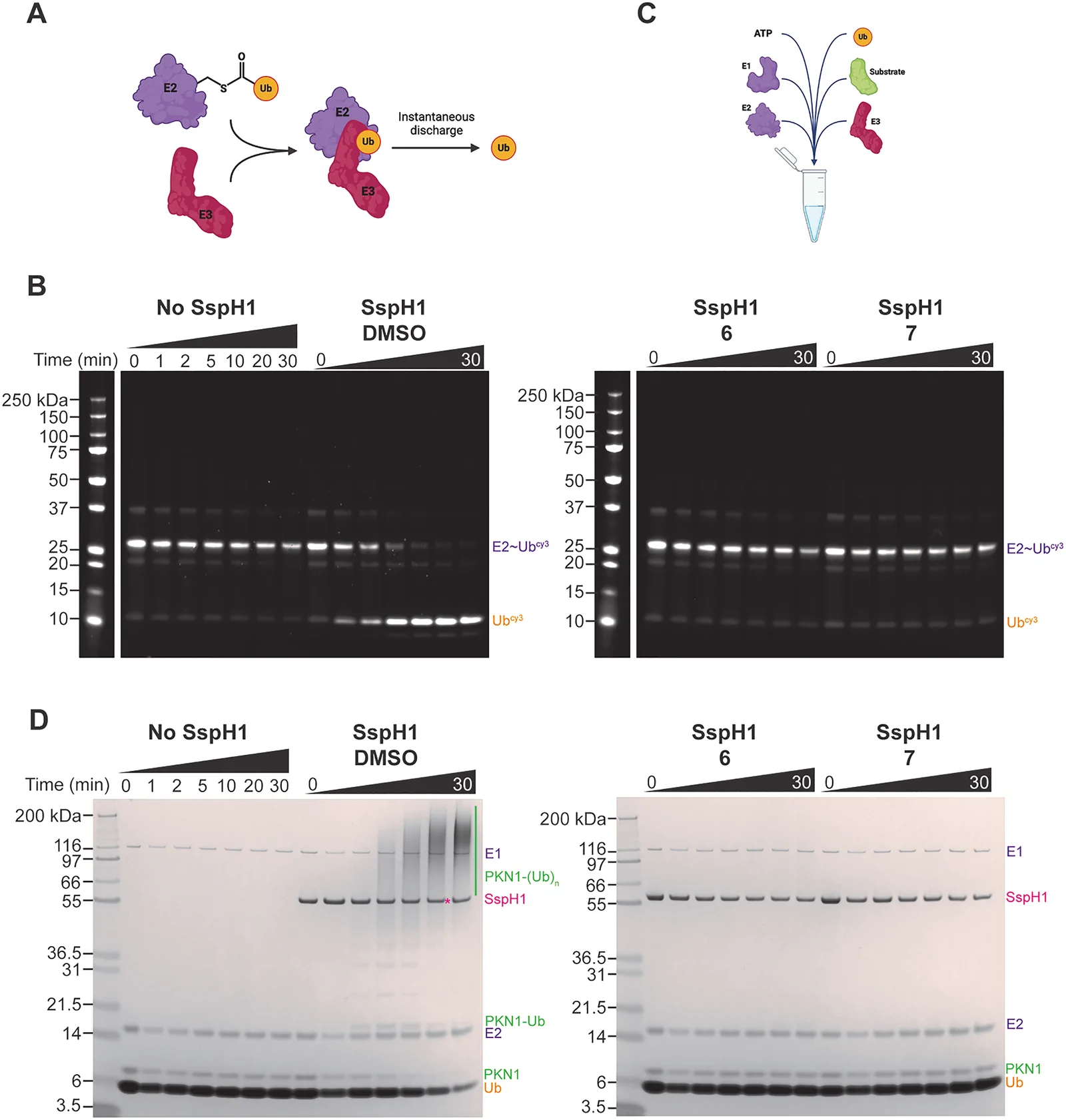
SspH1 *in vitro* inhibition with compounds 6 and 7. (A) Discharge assay schematic; (B) E2–Ub discharge time course assay with SspH1 pre-treated with either DMSO or compounds 6 or 7. UbcH5A–Ub-cy3 (1 μM) was incubated at RT for 0–30 minutes with SspH1 (residues 161–700, 50 nM) which had been pre-labelled with 6 or 7 at RT overnight. (C) Substrate ubiquitination assay schematic; (D) PKN1 ubiquitination time course assay with SspH1 pre-treated with either DMSO or compounds 6 or 7. A reaction of UBA1 (0.1 μM), UbcH5A (2 μM), ubiquitin (20 μM), PKN1 HR1b (2 μM, residues 122–199) and 10 mM ATP was incubated at RT for 0–30 minutes with SspH1 (residues 161–700, 0.5 μM) which had been pre-labelled with 6 or 7 at RT overnight.

We next wanted to understand whether compounds 6 and 7 interfered with SspH1 E3 activity and turned to *in vitro* assays to assess E3 ligase activity. In the absence of substrate, NEL E3 ligases non-productively discharge ubiquitin from the E2–Ub thioester, a reaction that proceeds *via* an unstable E3-thioester intermediate.^27^ This activity can be followed by E2–Ub discharge assays (Fig. 4A). We compared discharge activity of SspH1 with UbcH5A–Ub-cy3 (E2–Ub*) pre-treated with a DMSO control or compounds 6 or 7 (Fig. 4B). To ensure complete compound labelling, SspH1 was pre-incubated with the compounds overnight at room temperature, as opposed to at 4 °C which enabled us to study kinetics previously. SspH1 discharge activity was completely abrogated by treatment with 6 or 7, indicating that the catalytic cysteine is blocked following compound treatment.

Alternatively, substrate ubiquitination can be followed *in vitro* using a reconstituted ubiquitin enzymatic cascade, where recombinant ubiquitin, E1, E2, E3 and substrate are incubated with ATP (Fig. 4C). With SspH1, E3 ligase activity can then be observed by ubiquitination of its substrate PKN1.^25^ This assay represents a higher order of complexity and involves three enzymes with catalytic cysteines (E1, E2 and E3) and is closer to *in situ* E3 activity. We compared SspH1 ubiquitination activity when SspH1 was pre-treated with DMSO or compound 6 or 7 (Fig. 4D), again overnight at room temperature to ensure complete labelling. Prior to starting the assay, the pre-treated SspH1 mixture was significantly diluted to prevent E1 or E2 labelling with either compound. Furthermore, we had previously demonstrated that UbcH5A is not labelled by compound 6 or 7 (Fig. 3C and D), however the large size of E1 precludes accurate deconvolution by intact protein LC-MS. PKN1 ubiquitination was completely abrogated by treatment with 6 or 7, providing further evidence that SspH1 is completely inhibited by these compounds.

### Lysate engagement with SspH1 and SspH2 with compound 6 and 7

To understand whether compound 6 or 7 would be a useful starting point for tool compound development for SspH1 and/or SspH2, we interrogated whether the compounds could engage these targets in a cellular context using chemoproteomics. We first assessed compound labelling of the catalytic cysteines using human cell lysate spiked with recombinant SspH1 (C492) and SspH2 (C580).

**Fig. 5:**
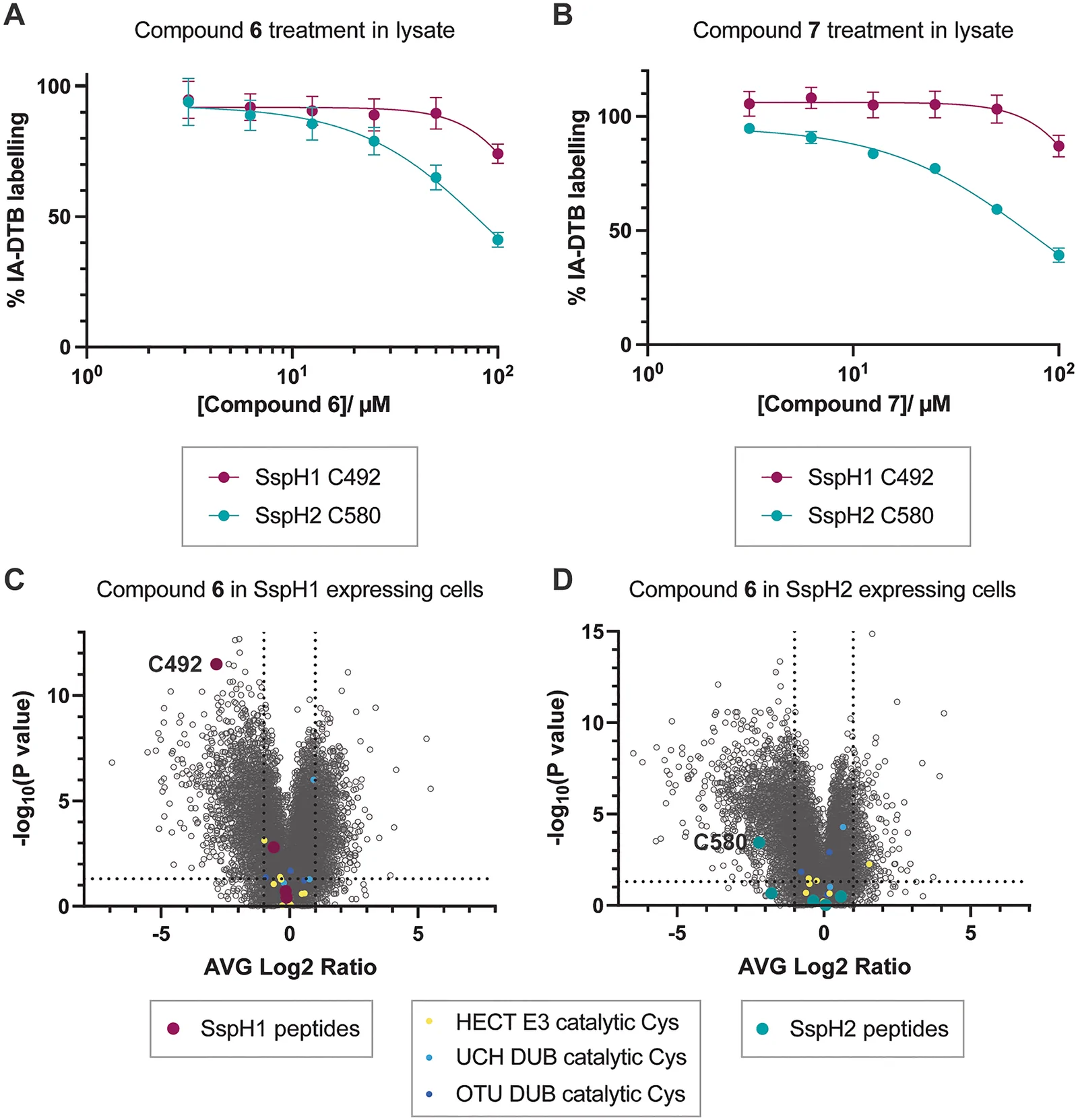
Characterisation of protein interactions by compounds 6 and 7. (A) SspH1 and SspH2 catalytic cysteine labelling with compound 6 (100–3.125 μM, 4 hours) in HEK293T lysate spiked with recombinant SspH1 and SspH2. Data are presented as mean ± SEM, *n* = 4. The curves were fitted with GraphPad Prism 10 using four parameter nonlinear regression with baseline correction to DMSO-treated samples. Full volcano plots for each condition are shown in Fig. S5A. (B) SspH1 and SspH2 catalytic cysteine labelling with compound 7 (100–3.125 μM, 4 hours) in HEK293T lysate spiked with recombinant SspH1 and SspH2. Data are presented as mean ± SEM, *n* = 4. The curves were fitted with GraphPad Prism 10 using four parameter nonlinear regression with baseline correction to DMSO-treated samples. Full volcano plots for each condition are shown in Fig. S5B. (C) Volcano plot of IA-DTB competition in SspH1 expressing HEK293T cells treated with compound 6 (50 μM, 4 hours). (D) Volcano plot of IA-DTB competition in SspH2 expressing HEK293T cells treated with compound 6 (50 μM, 4 hours). Data is shown as compared to DMSO treated samples, with competed peptides in the upper left-hand quadrant. Competed catalytic cysteine-containing peptides of HECT E3s (yellow), UCH DUBs (blue) and OTU DUBs (purple) are indicated. All proteomics experiments were performed with technical quadruplicates.

HEK293T lysate supplemented with recombinant SspH1 (161–700) and SspH2 (166–783) was treated with a dilution series (50–1.56 μM) of either compound 6 or 7 for four hours at RT. Following compound treatments, we used an iodoacetamide-desthiobiotin (IA-DTB) competitive chemoproteomics workflow to assess cysteine engagement.^52^ Comparison of DMSO treated lysate with compound treated lysate enabled identification of peptides where IA-DTB labelling of cysteines was blocked due to fragment engagement (Fig. S5). We were pleased to observe concentration-dependent competition of IA-DTB labelling of both the C492 peptide for SspH1, and the C580 peptide for SspH2 with both compounds 6 and 7 (Fig. 5A and B). Competition of the SspH2 C580 peptide occurred at lower concentrations compared to the SspH1 C492 peptide, which corroborated our previous kinetics experiments that both compounds label SspH2 faster than SspH1 (Fig. S4). Furthermore, this experiment also indicated that compounds 6 and 7 are promiscuous protein labellers in human cell lysate, with high numbers of engaged peptides (identified with an average log_2_ competition ration (CR) ≤−1 and *P*-value ≤0.05 when compared to DMSO controls) (Fig. S5), suggesting that further medicinal chemistry optimisation would be required to turn either compound into a specific inhibitor of SspH proteins.

### In cell engagement of SspH1 and SspH2 with compound 6

We next interrogated whether compounds 6 or 7 could engage SspH1 or SspH2 in live human cells. To simplify experimental setup, we opted for NEL overexpression in mammalian cells, coupled with chemoproteomics, over a *Salmonella* infection-based assay. SspH1 or SspH2 were transiently expressed in HEK293T cells (Fig. S6), and cells treated with 50 μM of either compound 6 or 7 for four hours. We were unable to collect *in cellulo* proteomics data for compound 7 due to significant effects on cell attachment and potential toxicity. In contrast we observed no apparent cell toxicity with compound 6. Following cell lysis, we again utilized an IA-DTB chemoproteomics workflow to assess cysteine engagement.^52^ Peptides engaging with compound 6 were identified with an average log_2_ CR ≤ −1 and *P*-value ≤0.05 when compared to DMSO controls (Fig. 5C and D). We observed strong competition of IA-DTB labelling of the catalytic cysteine C492 peptide of SspH1 (Fig. 5C) and C580 peptide of SspH2 (Fig. 5D), with no other SspH1 or SspH2 peptides showing engagement with compound 6. Similarly to our lysate chemoproteomics experiments, we observed engagement of multiple mammalian proteins. However, none of these included catalytic cysteines-containing peptides of HECT or RBR E3 ligases, nor of DUBs. Therefore, compound 6 provides a useful starting point for medicinal chemistry campaigns to design potent and specific tool compounds for SspH1 and SspH2.

### Structural analysis of protein-compound complexes

**Fig. 6:**
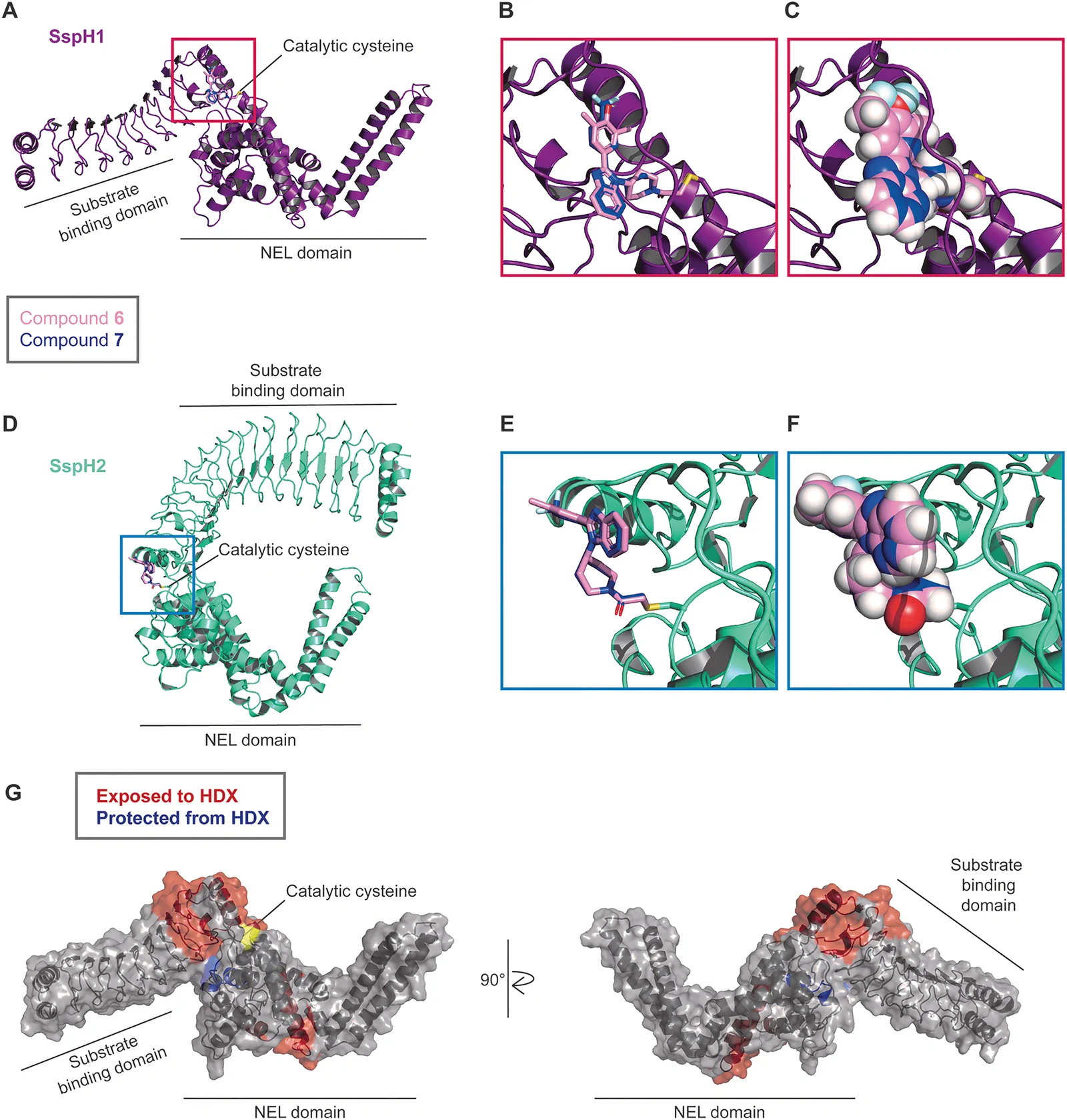
Characterisation of compound–protein interactions with structural biology. Molecular docking of 6 (pink) and 7 (blue) into X-ray structures of (A) SspH1 (PDB 9H6W), with (B) zoomed view of catalytic cysteine C492, and (C) space-filling view of fragments; and (D) SspH2 (PDB 3G06), with (E) zoomed view of catalytic cysteine C580, and (F) space-filling view of fragments. Interaction maps can be found in Fig. S7. (G) HDX-MS data depicted on SspH1 structure (PDB 9H6W), with areas protected from solvent exchange upon compound labelling shown in blue, and those with increased solvent exchange shown in red. Full HDX-MS data can be found in Fig. S8 and S9.

To better understand the interactions of compounds 6 and 7 with SspH proteins we used structural methods to analyse compound engagement by the catalytic cysteines. Unfortunately, attempts to crystallise SspH1 with either compound were unsuccessful, and so we used covalent molecular docking (MOE) of X-ray crystal structures of SspH1 (PDB 9H6W^30^) and SspH2 (PDB 3G06^24^) to respective catalytic cysteines C492 and C580 (Fig. 6A–F and Fig. S7). Intriguingly, the two SspH proteins adopt different conformations in crystal structures, with both conformations shown to exist in solution for SspH1,^30^ providing us with an opportunity to explore how these compounds might affect interdomain dynamics.

Both compounds 6 and 7 engage the catalytic cysteine, which is located at the interface between the LRR and NEL domains of SspH1 (Fig. 6A–C), and of SspH2 (Fig. 6D–F). Of note, the ‘closed’ conformation captured by the SspH2 crystal structure does not have a well-defined binding pocket close to the catalytic cysteine, whereas the ‘open’ conformation of SspH1 does. In the ‘open’ conformation represented by the SspH1 structure, the dimethyl phenol group of 6 and the trifluoro benzyl group of 7 are directed into a pocket, whereas the benzimidazole group is solvent exposed (Fig. 6B, C and Fig. S7A, B). In the ‘closed’ conformation represented by the SspH2 structure, there are fewer predicted interactions between protein and compound and more solvent exposure (Fig. S7C and D). This result reflects differences in protein–compound interactions in the two distinct protein conformations that have been trapped in the crystal structures and may not accurately represent the binding mode in solution where SspH2 is likely to show a similar conformational flexibility as SspH1. Therefore, we cannot infer conclusions from these predictions about interactions that lead to faster labelling kinetics with SspH2 over SspH1. However, we hypothesised that by binding at this interdomain protein–protein interaction site, compounds 6 and 7 might act as an internal SspH molecular glue, stabilising the ‘open’ conformation captured by the crystal structure of SspH1 over the ‘closed’ conformation captured by the SspH2 structure.

To better understand the dynamics of SspH1 upon compound binding, and to give greater confidence to our molecular docking predictions, we applied in-solution structural techniques. We first used hydrogen–deuterium exchange mass spectrometry (HDX-MS) to compare apo SspH1 and SspH1 labelled with either compound 6 or 7 (Fig. S8 and S9). We observed near to complete agreement between perturbations when liganded by 6 and 7, confirming that both compounds share the same binding mode with SspH1. In addition, we observed distinct areas of exposure and protection from HDX in compound labelled protein. When mapped onto the SspH1 structure, we observed an area close to our predicted docking pocket that became protected by compound engagement (Fig. 6G, shown in blue), building confidence in our docking predictions. Furthermore, some areas of the protein become more exposed upon compound labelling (Fig. 6G, shown in red), suggesting that compound binding induces changes in the solvent accessibility of protein surfaces further away from the active site.

We further used small angle X-ray scattering (SAXS) to compare conformational flexibility of apo SspH1 and SspH1 labelled with either compound 6 or 7 (Fig. S11 and Table S3). We observed that all three conditions gave near to identical values for maximum dimension (*D*_max_) and radius of gyration (*R*_g_) (Table S3), while the protein flexibility as assessed by dimensionless Kratky plots is unchanged upon compound treatment (Fig. S11). These data indicate that compounds 6 or 7 do not induce major changes in the conformational dynamics of SspH1.

## Conclusion

Currently major international efforts target developing chemical probes for the entire human proteome, while chemical probes are missing for many bacterial proteins that affect human health and disease. Multiple structural and mechanistic studies have provided insight into NEL bacterial E3 ligases,^24,27,29–31,53^ however no tool compounds have been described. In this study we utilised reactive fragment-based screening with a library of 227 chloroacetamides to discover cysteine reactive covalent ligands with the aim of inhibiting the catalytic activity of NEL effector proteins. Screening against *Shigella* IpaH9.8 and *Salmonella* SspH1 revealed key differences in their ligandability, and we identified three hit fragments against SspH1 for further development.

For rapid fragment elaboration, we deployed a high throughput chemistry direct-to-biology platform to screen 430 structurally related chloroacetamides against SspH1. Several of our HTC compounds fully labelled our target protein SspH1. To better understand the potency of these compounds we performed a concentration response labelling experiment, and selected our two best compounds, 6 and 7, for further follow up. We observed that 6 and 7 showed little to no reactivity with recombinant human ubiquitin binding proteins, nor with *Shigella* NEL IpaH9.8. Furthermore, we show that 6 and 7 were also potent ligands for SspH2, potentially paving the way for development of a pan-SspH tool compound.

We next tested whether compounds 6 or 7 were able to block SspH1 activity *in vitro*. We performed both E2–Ub discharge and substrate ubiquitination assays with SspH1, and observed complete abrogation of E3 ligase activity upon SspH1 pre-treatment with either compound. We further demonstrated target engagement of compound 6 and 7 with SspH1 and SspH2 in cell lysates, and compound 6 in live mammalian cells, using an IA-DTB chemoproteomics workflow. Combining molecular docking with in-solution structural biology using HDX-MS and SAXS revealed how compounds 6 and 7 likely interact with SspH1 and SspH2.

Compound 6 now represents a useful starting point for a medicinal chemistry campaign to develop a potent and selective pan-SspH inhibitor and tool compound. It is now, more than ever, imperative that tool molecules are developed against novel bacterial targets to better understand their role in infection and to identify therapeutic targets in the race against antimicrobial resistance.

## Materials and methods

Chloroacetamide fragment library information can be found in SI, along with supplier codes for compounds 1–10.

### Protein expression and purification

Proteins were expressed in *E. coli* BL21 cells (Agilent Technologies, Cat# 230132) with N-terminal His-tags, and isolated using nickel affinity purification following lysis by sonication. Following tag removal by cleavage with 3C protease, proteins were purified by gel filtration and stored at −80 °C in 50 mM HEPES pH 7.5, 150 mM NaCl, 0.5 mM TCEP until needed. The following proteins were used in this study:

**Table:** 

Protein	Construct
SspH1	161–700
SspH1 C492K	161–700
IpaH9.8	21–545
SspH2	166–788
PKN1 HR1b	122–199
UBA1 (E1)	1–1058
UbcH5A (E2)	1–147
Ubiquitin	1–76
HOIL	1–510
OTUD4	1–156
OTUD5	172–351
UBE3C	693–1083

### Round 1 screening

1 μM IpaH9.8 or SspH1 were incubated with 200 μM fragments for 24 hours at 4 °C, in 25 mM HEPES pH 7.5, 50 mM NaCl buffer. Intact protein LC-MS was performed as previously described.^40^ The following deconvolution conditions were used for recombinant proteins studied in this work:

**Table:** 

Protein construct	Expected mass range	*m*/*z* range
IpaH9.8 aa 21–545	58 000–62 000	350–2000
SspH1 aa 161–700	58 000–62 000	350–2000

### High throughput chemistry direct-to-biology (HTC-D2B) screening

HTC libraries of parent amines were designed by using the parent amine SMILES strings of compounds 1–3 as inputs for structural similarity search. Structurally similar amines were searched within GSK solution and solid stocks, using criteria 110 < MW < 350, primary and/or secondary aromatic amines excluded, and phenols and tricyclic compounds excluded. Resulting amines were plated as 10 mM stock solutions in DMSO (20 μL, 1 eq.) in a 384-well plate. To each well containing amine, a solution of *N*-(chloroacetoxy)succinimide (2 eq.) and *N*,*N*-diisopropylethylamine (3 eq.) in DMSO (20 μL) was added, mixed by pipetting and left to incubate for one hour. A column of DMSO only controls, and reagent only controls was also dispensed on the 384-well plate. Following reaction, an aliquot of each reaction mixture (diluted to 2.22 mM) was analysed by LC-MS. Immediately prior to incubation with proteins, each reaction mixture was quenched with hydroxylamine (100 μM). 1 μM IpaH9.8 or SspH1 were incubated with 50 μM HTC-D2B library for 24 hours at 4 °C, in 25 mM HEPES pH 7.5, 50 mM NaCl buffer. Intact protein LC-MS was performed as described above.

### Compound synthesis

Compounds 4–10 were purchased from Enamine (catalogue numbers in Table S2). Upon arrival, compound purities were confirmed as >90% by LC-MS and ^1^H NMR.

### Concentration validation experiments

Dilution series experiments were performed as for 1st round screening. 1 μM OTUD4, OTUD5 or 0.5 μM HOIL, UbcH5A, SspH2, SspH1 C492K were incubated with 100–6.25 μM compounds for 24 hours at 4 °C, in 25 mM HEPES pH 7.5, 50 mM NaCl buffer. Intact protein LC-MS was performed as previously described.^40^ The following deconvolution conditions were used for recombinant proteins studied in this work:

**Table:** 

Protein construct	Expected mass range	*m*/*z* range
OTUD4 aa 1–156	16 000–20 000	350–2000
OTUD5 aa 172–351	19 000–23 000	350–2000
SspH1 C492K aa 161–700	58 000–62 000	350–2000
SspH2 aa 166–783	66 000–70 000	350–2000
HOIL aa 1–510	56 000–60 000	350–2000
UbcH5A aa 1–147	15 000–19 000	350–2000

### Full kinetics characterisations

Recombinant SspH1 (aa 161–700) and SspH2 (aa 166–783) were both characterised against compounds 6 and 7. A dilution series in DMSO was prepared for the compound, and 1 μL added to three separate wells in a 384 well plate, representing technical triplicates of each condition. 99 μL of 0.5 μM SspH1 or SspH2 in 25 mM HEPES pH 7.5, 50 mM NaCl buffer was added to the wells and mixed thoroughly (final compound concentrations 100, 50, 25, 12.5, 6.25, 3.125, 1.56 μM). This mixture was then dispensed into 8 wells of 10 μL each in a new 384 well plate, one for each time point. The plate was incubated at 4 °C during intact MS. Intact protein LC-MS was performed as previously described,^40^ at approximate time points 0, 1, 2, 4, 6, 8 and 12 hours, and deconvoluted as above. The exact times of each measurement were saved with each reading and used for kinetics calculations. Labelling percentages were plotted against time in GraphPad Prism v.10, and curves fitted separately for each replicate using one-phase association, with constraints *Y*_0_ = 0 and plateau = highest labelling percentage. Rate constants (*k*_obs_, as given by Graphpad Prism calculated *K* values) were then plotted against concentration in triplicate, and straight lines fitted with constraint *Y*_intercept_ = 0. Data are presented as mean ± SD, *n* = 3. Slope values were converted from μM^−1^ hour^−1^ to M^−1^ s^−1^ to give *k*_inact_/*K*_I_ values. For SspH2 kinetics, 100 μM *k*_obs_ were not used to calculate *k*_inact_/*K*_I_, as they were outside the linear range. Reported errors are standard error, as calculated in GraphPad Prism v.10.

### Glutathione reactivity assays

10 mM DMSO stocks of compounds 6 and 7 were diluted 20-fold with acetonitrile, and then further 5-fold diluted with 6 mM glutathione in PBS. The reaction was shaken before incubation at 40 °C. The reaction mixture was analysed *via* UPLC-UV-MS up to eight times across 24 hours, compared to known reference compounds and samples of each compound in distilled water. UPLC conditions: flow rate: 800 μL min^−1^; column: acquity UPLC BEH C18 1.7 μm 2.1 × 50 mm; column temperature: 37 °C; mobile phase A: 0.1% formic acid in H_2_O; mobile phase B: 0.1% formic acid in 100% ACN; run time: 2 minutes; gradient elution: 97% A to 0% A; UV conditions: 210 to 350 nm range; MS conditions: single quad, ESI+, scan range: 50–1000 Da. For each time point, the UV peak area of the parent peak was extracted at a single wavelength (*e.g.*, 254 nm). A pseudo-first-order rate constant (*k*) for each compound was determined from the slope of a linear regression fit for a plot of the logarithm base-10 peak area of the parent compound *versus* the time differential for the eight time points; the *t*_1/2_ is calculated as follows: *t*_1/2_ = 0.693/*k*.

### *In vitro* inhibition assays

#### E2–Ub discharge assay

SspH1 (2.5 μM, 161–700) was prelabelled at 350 rpm, RT for 18 hours overnight with 50 μM compound 6 or 7 in 50 mM HEPES pH 7.5, 150 mM NaCl, 1% DMSO. UbcH5A–Ub-cy3 (1 μM) was incubated at RT for 0–30 minutes with pretreated SspH1 (50 nM) in 50 mM HEPES pH 7.5, 150 mM NaCl. Time points were quenched with sample loading dye (Invitrogen NuPAGE) and snap frozen in liquid nitrogen. All samples were thawed and run by SDS-PAGE with a fluorescent ladder (LI-COR Molecular Weight Marker 928-40000) on 4–12% gels (Invitrogen NuPAGE) at 200 V for 30 minutes. Gels were fluorescently imaged for cy3 (for ubiquitin) and AlexaFluor647 (for ladder).

#### Substrate ubiquitination assay

SspH1 (5 μM, 161–700) was prelabelled at 350 rpm, RT for 18 hours overnight with 100 μM compound 6 or 7 in 50 mM HEPES pH 7.5, 150 mM NaCl, 1% DMSO. A reaction of UBA1 (0.1 μM), UbcH5A (2 μM), ubiquitin (20 μM), PKN1 (2 μM, 122–199) with pretreated SspH1 (50 nM) in 25 mM HEPES pH 7.5, 150 mM NaCl, 10 mM MgCl_2_, 0.5 mM TCEP was initiated with the addition of 10 mM ATP. The reactions were incubated at RT for 0–30 minutes, and time points quenched with sample loading dye (Invitrogen NuPAGE) containing DTT before being snap frozen in liquid nitrogen. All samples were thawed, heated at 95 °C for 3 minutes, and run by SDS-PAGE with Mark12 unstained ladder (Invitrogen) on 4–12% gels (Invitrogen NuPAGE) at 200 V for 30 minutes. Gels were stained with quick Coomassie stain (Protein Ark), washed in water and imaged.

### Target engagement in lysates

A pellet of 50 × 10^6^ HEK293T cells was resuspended in 5 mL lysis buffer (50 mM HEPES pH 8.0, 150 mM NaCl, 1% IGEPAL, 0.1% SDS, 0.5% Na-deoxycholate, supplemented with protease inhibitor cocktail (Sigma-Aldrich, P8340)). The lysate was sonicated and centrifuged (10 min, 17 000 × *g*) and the soluble fraction isolated. Protein concentrations were measured using a Rapid BCA Gold Protein Assay Kit (Thermo Scientific, A53226) and diluted to 0.75 mg mL^−1^ with lysis buffer. The lysate was then spiked with 0.2 μM of each recombinant SspH1 (aa 161–700) and SspH2 (aa 166–783). For each condition, four wells were treated and processed separately, representing four technical replicates. 2 μL of 100× stocks of compounds 6 and 7 in DMSO (10–0.156 mM, and DMSO only control), and 200 μL lysate was added to a 96-well plate. The plate shaken at room temperature for 4 hours. Samples were prepared for IA-DTB chemoproteomics as described below.

### *In cellulo* target engagement

All cells were grown in DMEM media (Gibco, 41966-029) supplemented with FBS (ThermoFisher, A5256701) and Pen/Strep (Gibco, 15140-122) at 37 °C with 5% CO_2_. SspH1 (aa 1–700) and SspH2 (aa 1–786) genes were cloned separately into pcDNA1.3-FLAG plasmids. HEK293T cells were seeded in 6-well plates at a density of 2 × 10^5^ cells per well. For each condition, four wells were treated and processed separately, representing four technical replicates. After 24 hours, cells were transfected with 500 ng DNA with 1.5 μL 1 mg mL^−1^ polyethylenimine (PEI, Sigma, 764965) in 1 mL Opti-MEM (ThermoFisher, 31985062) per well for 4 hours, before replacing with normal media. After 24 hours, the cells were washed with PBS and treated with either DMSO, 50 μM compound 6 or 50 μM compound 7 in 1 mL media for 4 hours. For all conditions the DMSO concentration was 0.1%. Cells were washed three times with PBS and lysed in 200 μL lysis buffer (50 mM HEPES pH 8.0, 150 mM NaCl, 1% IGEPAL, 0.1% SDS, 0.5% Na-deoxycholate, supplemented with protease inhibitor cocktail (Sigma-Aldrich, P8340)) per well. Lysates were sonicated and centrifuged (10 min, 17 000 × *g*) and the soluble fraction isolated. Protein concentrations were measured using a Rapid BCA Gold Protein Assay Kit (Thermo Scientific, A53226) and diluted to 1 mg mL^−1^ with lysis buffer. Samples were prepared for IA-DTB chemoproteomics as described below.

### IA-DTB chemoproteomics

Lysates were treated with 500 μM iodoacetamide-desthiobiotin (IA-DTB) at room temperature (RT) for 1 hour, shaken at 700 rpm, and then reduced with 5 mM dithiothreitol (DTT) under the same conditions for 30 minutes, and finally alkylated with 10 mM iodoacetamide (IAA) for 30 minutes. Proteins were precipitated with the addition of 5 mg of glass spheres (Sigma Aldrich, 440345) in 800 μL acetonitrile (MeCN) and shaken at 600 rpm for 5 minutes at RT. Beads were washed three times with 80% MeCN and centrifuged to remove any remaining solvent. The beads were shaken overnight at RT with 2 μg of trypsin (Thermo Scientific, 90059) in 250 μL 50 mM HEPES pH 8.5.

Peptides were isolated by filtering and washing the glass beads with 50 mM HEPES pH 8.5 and collecting the flow-through. Peptides were then incubated with 50 μL neutravidin beads in 50 mM HEPES pH 8.5 for 2 hours at RT, 1000 rpm. Beads were washed three times with each of the following: 0.1% SDS in 50 mM HEPES pH 8.5; 50 mM HEPES pH 8.5; proteomics-grade water. Peptides were then eluted from the beads in a total of 400 μL 0.1% formic acid in 50% MeCN/water, and lyophilised.

Peptides were redissolved in 100 μL 0.1% formic acid in water, before loading the samples and iRT standard onto Evotips, which were prepared according to manufacturer's instructions. The peptides were analysed using an Evosep One LC system coupled with a timsTOF Pro 2 mass spectrometer *via* a CaptiveSpray nano-electrospray ion source. Data for all samples was acquired in diaPASEF mode using the 60 SPD predefined method on Evosep One, which was fitted with an 8 cm column (EV1109). Mobile phase A was 0.1% formic acid in water and mobile phase B 0.1% formic acid in acetonitrile.

For all samples, mass spectra were acquired from 100–1700 *m*/*z*. The ion mobility range was set to 0.6–1.60 V s cm^−2^. TIMS accumulation and ramp times were set to 100 ms. 12 diaPASEF scans were collected per one TIMS-MS scan, giving a duty cycle of 1.37 s. 24 variable mass and mobility windows were set over the mass range 400–1399.8 *m*/*z* and mobility range 0.6–1.60 V s cm^−2^ (table below). The collision energy was increased linearly from 20 eV to 59 eV between 0.6–1.60 V s cm^−2^.

#### diaPASEF isolation windows

**Table:** 

#MS type	Cycle Id	Start IM [1/*K*_0_]	End IM [1/*K*_0_]	Start mass [*m*/*z*]	End mass [*m*/*z*]
MS1	0	—	—	—	—
PASEF	1	0.91	1.6	757.73	781.38
PASEF	1	0.6	0.91	400.19	497.58
PASEF	2	0.94	1.6	781.38	805.41
PASEF	2	0.6	0.94	497.58	538.28
PASEF	3	0.96	1.6	805.41	832.41
PASEF	3	0.6	0.96	538.28	564.8
PASEF	4	0.98	1.6	832.41	858.99
PASEF	4	0.6	0.98	564.8	586.29
PASEF	5	0.99	1.6	858.99	889.12
PASEF	5	0.6	0.99	586.29	607.62
PASEF	6	1.01	1.6	889.12	922.45
PASEF	6	0.6	1.01	607.62	628.3
PASEF	7	1.02	1.6	922.45	957
PASEF	7	0.6	1.02	628.3	648.3
PASEF	8	1.04	1.6	957	996.96
PASEF	8	0.6	1.04	648.3	669.71
PASEF	9	1.06	1.6	996.96	1044.05
PASEF	9	0.6	1.06	669.71	691.85
PASEF	10	1.08	1.6	1044.05	1106.52
PASEF	10	0.6	1.08	691.85	713.34
PASEF	11	1.12	1.6	1106.52	1195.59
PASEF	11	0.6	1.12	713.34	735.02
PASEF	12	1.19	1.6	1195.59	1399.75
PASEF	12	0.6	1.19	735.02	757.73

The data was searched using Pulsar search engine in Spectronaut (v. 18.7.240506.55695) against human uniprot (Oct 2022), D0ZVG2_SspH1, D0ZPH9_SspH2 and universal contaminants fasta files using directDIA method. IA-DTB (C14H24O3N4, 296.18 Da) and carbamidomethyl were selected as variable modifications for cysteine residue. PTM workflow and localisation filter were selected. The data was normalised using global median normalisation strategy with automatic row selection. Modified sequence was selected for minor (peptide) grouping. Other search settings were used as default (BGS factory settings). Unpaired *t*-test was used to determine average log 2 ratios (fragment/DMSO) and *p*-values for IA-DTB labelled peptides. Volcano plots and concentration-dependent labelling curves made in GraphPad Prism v.10. The mass spectrometry proteomics data have been deposited to the ProteomeXchange Consortium *via* the PRIDE partner repository with the dataset identifier PXD057304.^54,55^

### Transfection optimisation and western blot

HEK293T cells were plated and transfected as above, with the indicated quantities of DNA per well of a 6-well plate. 24 hours after transfection each well was lysed in 30 μL lysis buffer (0.5% IGEPAL, 150 mM NaCl, 50 mM Tris pH 7.5, 5 mM MgCl_2_, cOmplete mini EDTA-free Protease Inhibitor Cocktail (Merck, 4693159001) at 4 °C, then lysates centrifuged 14 500 × *g*, 10 min, 4 °C. 10 μL 4× SDS loading buffer (Invitrogen, NP0007) was added to the resulting supernatants before boiling briefly at 95 °C. Samples were then run on NuPAGE 4–12% Bis-Tris gels (Invitrogen) alongside PageRuler Prestained Plus molecular weight ladder (ThermoFisher), following which samples were transferred onto nitrocellulose membranes using the BioRad TransBlot Turbo system as per the manufacturer's instructions. Membranes were blocked in 5% milk in PBS with 0.1% Tween (PBST) for 30 min. Primary antibodies (anti-FLAG-HRP (Merck, A8592) or anti-GAPDH (Millipore, MAB347)) were incubated in 5% milk/PBST RT for 2 hours, before washing with PBST and incubating with anti-mouse-HRP (Cell Signaling, #7074) for 1 hour. Finally, membranes were washed with PBST and then developed using chemiluminescence detection reagents (Amersham, RPN2106), with images taken on BioRad ChemiDoc and assembled in ImageLab software.

### Molecular docking

SspH1 and SspH2 crystal structures (PDB 9H6W^30^ and 3G06^24^) were imported into Molecular Operating Environment 2020.0901 (Chemical Computing Group, Montreal, Canada), and prepared using the in-built ‘QuickPrep’ function (default parameters). The covalent docking protocol implemented in MOE was employed to generate docking conformations of compounds 6 and 7, attached to active site cysteines using the ‘alpha_halocarbonyl_S’ reaction. Refinement was carried out using the rigid receptor method, based on the GBVI/WSA dG scoring functionality, to give 5 final poses. The best poses identified by the docking were taken forward for further molecular analyses. Generation of a 2D ligand interactions map for the highest scoring docking pose was also performed within Molecular Operating Environment 2020.0901 (Chemical Computing Group, Montreal, Canada), using the ‘Ligand Interactions’ function. Figures of docked ligands were generated in PyMOL 2.3.1 (Schrödinger, LLC), using ligands in stick or sphere representation.

### Hydrogen deuterium exchange mass spectrometry (HDX-MS)

SspH1 (25 μM, 161–700) was mixed gently with compound 6 or 7 (100 μM) for 18 hours overnight at RT in 50 mM HEPES pH 7.5, 150 mM NaCl, 1% DMSO, before purification by gel filtration. Apo SspH1 (161–700) and SspH1 pre-treated with either compound 6 or 7 were incubated at 5 μM with 40 μL of D_2_O buffer at room temperature for 3, 30, 300 and 3000 seconds in triplicate. The labelling reaction was quenched by adding chilled 2.4% v/v formic acid in 2 M guanidinium hydrochloride and immediately frozen in liquid nitrogen. Samples were stored at −80 °C prior to analysis.

The quenched protein samples were rapidly thawed and subjected to proteolytic cleavage by pepsin followed by reversed phase HPLC separation. Briefly, the protein was passed through an enzymate BEH immobilized pepsin column, 2.1 × 30 mm, 5 μm (Waters, UK) at 200 μL min^−1^ for 2 min and the peptic peptides trapped and desalted on a 2.1 × 5 mm C18 trap column (Acquity BEH C18 Van-guard pre-column, 1.7 μm, Waters, UK). Trapped peptides were subsequently eluted over 11 min using a 5–43% gradient of acetonitrile in 0.1% v/v formic acid at 40 μL min^−1^. Peptides were separated on a reverse phase column (Acquity UPLC BEH C18 column 1.7 μm, 100 mm × 1 mm (Waters, UK). Peptides were detected on a cyclic mass spectrometer (Waters, UK) acquiring over a *m*/*z* of 300 to 2000, with the standard electrospray ionization (ESI) source and lock mass calibration using [Glu1]-fibrino peptide B (50 fmol μL^−1^). The mass spectrometer was operated at a source temperature of 80 °C with a spray voltage of 3.0 kV. Spectra were collected in positive ion mode.

Peptide identification was performed by MS^e^,^56^ using an identical gradient of increasing acetonitrile in 0.1% v/v formic acid over 12 min. The resulting MS^e^ data were analyzed using Protein Lynx Global Server software (Waters, UK) with an MS tolerance of 5 ppm.

Mass analysis of the peptide centroids was performed using DynamX software (Waters, UK). Only peptides with a score >6.4 were considered. The first round of analysis and identification was performed automatically by the DynamX software, however, all peptides (deuterated and non-deuterated) were manually verified at every time point for the correct charge state, presence of overlapping peptides, and correct retention time. Deuterium incorporation was not corrected for back-exchange and represents relative, rather than absolute changes in deuterium levels. Changes in H/D amide exchange in any peptide may be due to a single amide or a number of amides within that peptide. All time points in this study were prepared at the same time and individual time points were acquired on the mass spectrometer on the same day.

### Small angle X-ray scattering (SAXS)

SspH1 (25 μM, 161–700) was mixed gently with compound 6 or 7 (100 μM) for 18 hours overnight at RT in 50 mM HEPES pH 7.5, 150 mM NaCl, 1% DMSO, before purification by gel filtration. SEC-SAXS data were collected at the B21 beamline at Diamond Light Source (DLS, UK). Apo SspH1 (161–700) and SspH1 pre-treated with either compound 6 or 7 samples at 10 mg mL^−1^ in 50 mM HEPES pH 7.5, 150 mM NaCl and 0.5 mM TCEP were injected onto a Superdex 200 3.2 × 300 column and eluted at a flow rate of 0.075 mL min^−1^ at 15 °C with 3 s exposures. Frames were collected continuously during the fractionation of the proteins. Frames collected before the void volume were averaged and subtracted from the signal of the elution profile to account for background scattering. Data reduction, subtraction, and averaging within the SEC peak with constant *R*_g_ were performed using the software ScÅtterIV (https://www.bioisis.net). The scattering curves were analyzed using the package ATSAS and reported as function of the angular momentum transfer *q* = 4π/*λ* sin *θ*, where 2*θ* is the scattering angle and *λ* the wavelength of the incident beam. The statistics are reported in Table S3. The statistics for apo SspH1 agree with our previous experiment which was run at SOLEIL synchrotron on a different SEC column and different beamline.^30^

## Author contributions

C. R. K. expressed and purified proteins, performed library screening, HTC-D2B synthesis and screening, full kinetics characterisations, and developed and performed biochemical assays. K. A. M. synthesised an HTC-D2B library and performed *in silico* docking experiments. J. D.-F. and C. R. K. performed cell experiments. A. V. and C. R. K. performed proteomics experiments and data analysis. W. J. M. performed data analysis. D. E. performed SAXS analysis. S. M. and J. M. S. performed HDX-MS experiments. J. P. and J. B. advised and performed compound library management. K. R. and D. H. conceived the project, data analysis, funding acquisition and supervision. C. R. K. wrote the paper with input from all authors.

## Conflicts of interest

The authors declare no competing interests.

## Supplementary Material

CB-007-D5CB00177C-s001

CB-007-D5CB00177C-s002

## Data Availability

The mass spectrometry proteomics data have been deposited to the ProteomeXchange Consortium *via* the PRIDE partner repository with the dataset identifier PXD057304. Chloroacetamide fragment library information can be found in Supplementary information (SI), along with supplier codes for compounds 1–10. Supplementary information is available. See DOI: https://doi.org/10.1039/d5cb00177c.
