# Supplementary material for: Covalent fragment screening to inhibit the E3 ligase activity of bacterial NEL enzymes SspH1 and SspH2
Source: RSC Chem Biol. 2025 Oct 28;7(1):153–68. doi: 10.1039/d5cb00177c (PMC12584752; doi:10.1039/d5cb00177c)
Supplement: CB-007-D5CB00177C-s001 [file CB-007-D5CB00177C-s001.pdf]

Supplementary Information (SI) for RSC Chemical Biology.
This journal is © The Royal Society of Chemistry 2025

Covalent fragment screening to inhibit the E3 ligase activity of bacterial NEL enzymes SspH1 and SspH2

Cassandra R. Kennedy[1], Katherine A. McPhie[1], Aini Vuorinen[2], Jane Dudley-Fraser[1], Diego Esposito[1], Sarah Maslen[2], William J. McCarthy[1], Jonathan Pettinger[3], J. Mark Skehel[2], Jacob Bush[3], David House[3], Katrin Rittinger[1]

1 Molecular Structure of Cell Signalling Laboratory, The Francis Crick Institute, 1 Midland Road, London, NW1 1AT, United Kingdom.

2 Proteomics Science Technology Platform, The Francis Crick Institute, 1 Midland Road, London, NW1 1AT, UK.

3 Crick-GSK Biomedical LinkLabs, GSK, Gunnels Wood Road, Stevenage, Hertfordshire, SG1 2NY, United Kingdom.

Supplementary Information Contents

Supplementary Figures 1-10

Supplementary Table 1: chloroacetamide library information (in accompanying csv file)

Supplementary Table 2: HTC-D2B hit compound information (in accompanying csv file)

Supplementary Table 3: SAXS parameters

The mass spectrometry proteomics data have been deposited to the ProteomeXchange Consortium via the PRIDE partner repository with the dataset identifier PXD057304.

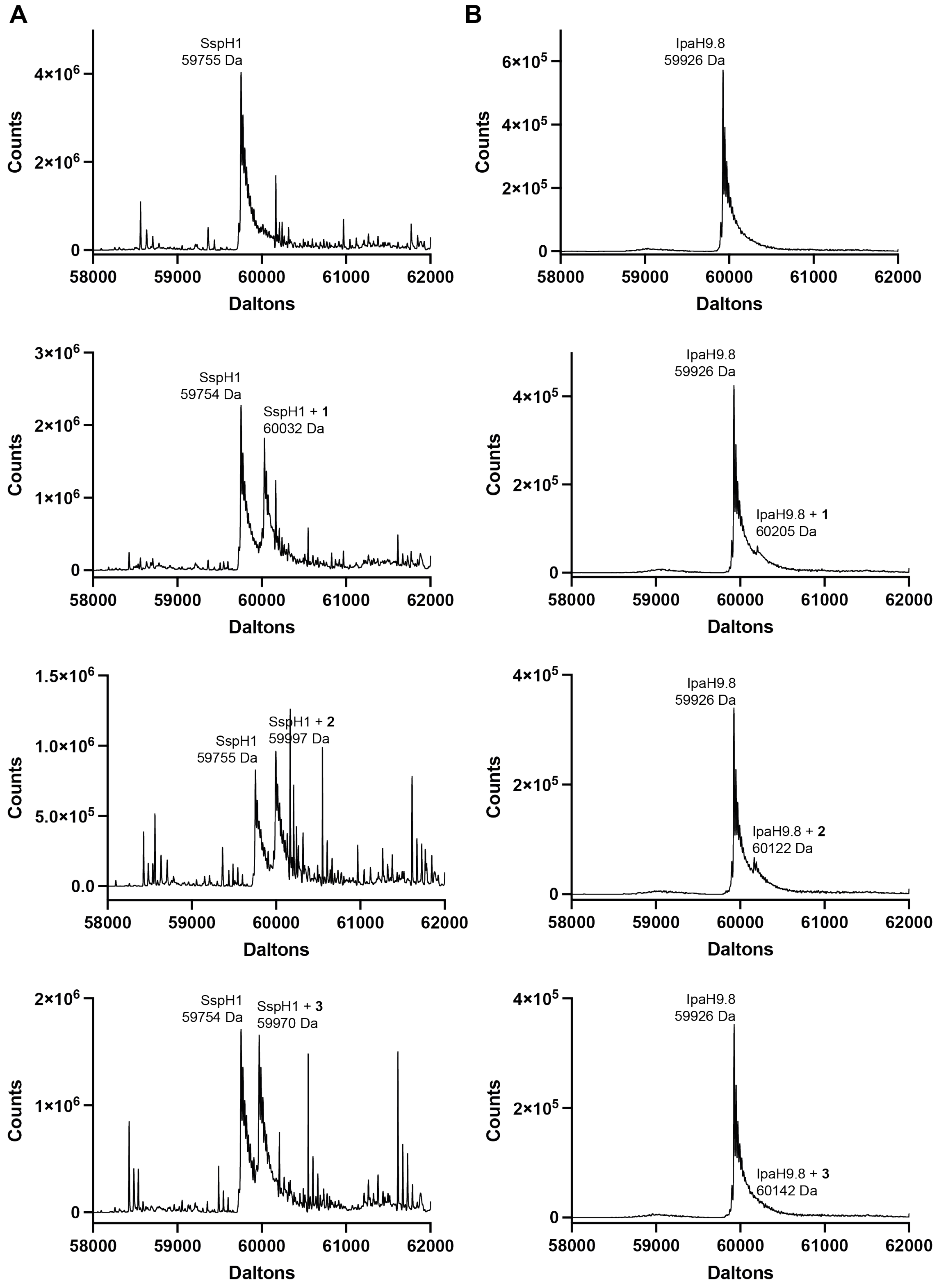

Supplementary Figure 1

Deconvoluted spectra for DMSO controls and fragments **1** - **3** labelling from round 1 screening against A) SspH1 and B) IpaH9.8. N.B high background noise on MS is visible in the SspH1 spectra, however the protein peaks were easily identifiable.

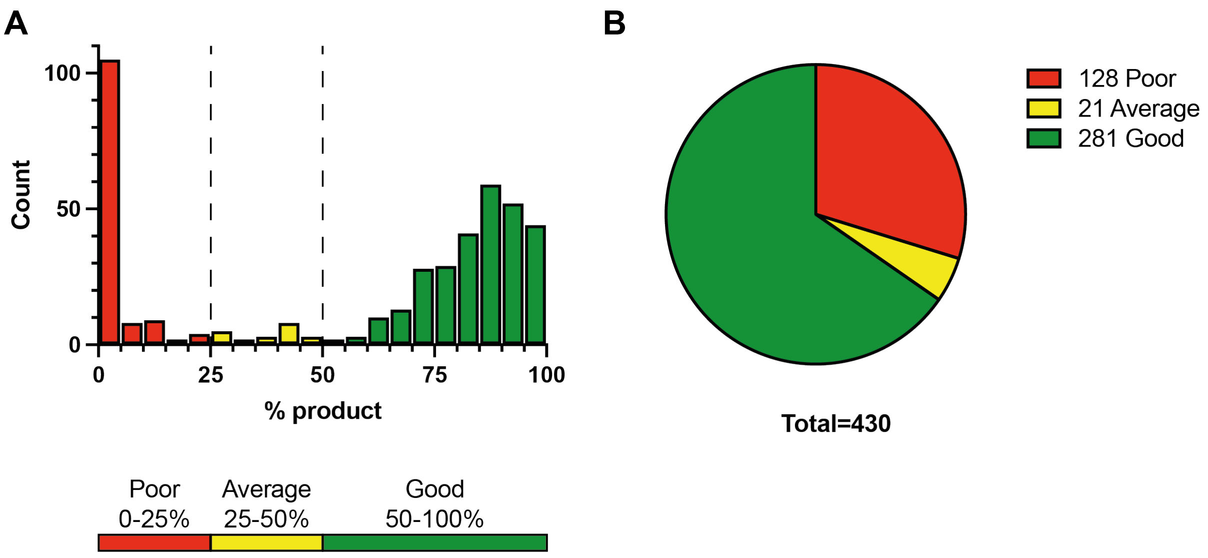

Supplementary Figure 2

High throughput chemistry conversion rates by LC-MS. Percentage of product was calculated from area under curve (AUC). A) Histogram of percentage conversions; B) Pie chart of conversion rates.

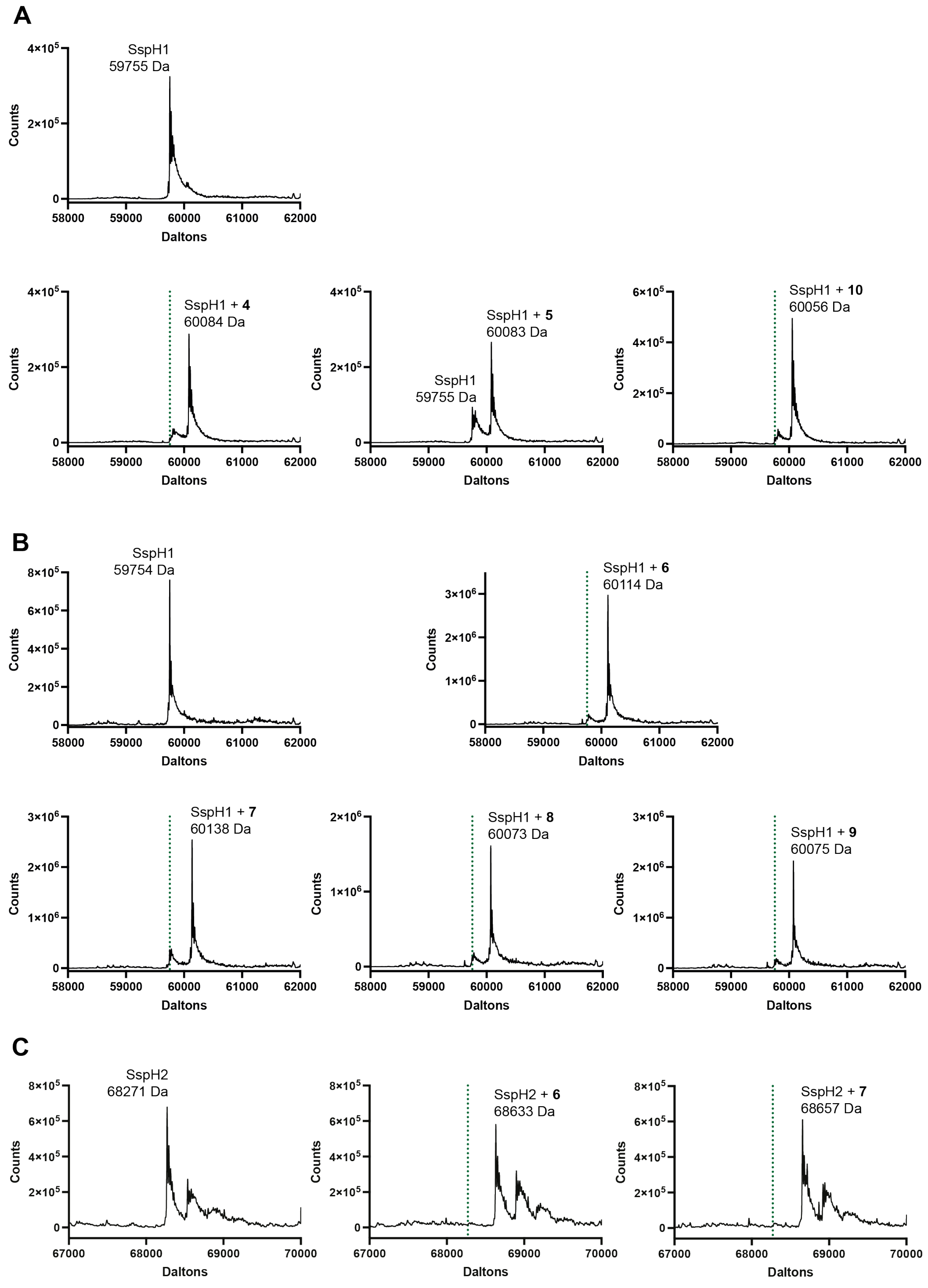

Supplementary Figure 3

Deconvoluted spectra for DMSO controls and compounds **4** - **10** labelling from HTC-D2B screening against SspH1. A) Compounds **4**, **5** and **10** from HTC plate 1 with their DMSO control; B) compounds **6** – **9** from HTC plate 2 with their DMSO control. C) deconvoluted spectra for DMSO controls and 50 μM compounds **6** and **7** labelling of SspH2 (detail from Figure 3C and D).

N.B. For compounds with very high labelling percentages, analysis scripts could not detect the correct peak for the unlabelled protein at MW 59755 or 59754 Da (for SspH1) or 68271 Da (for SspH2). For these compounds the position of this is given by the green dotted line on the deconvoluted spectra, and labelling percentages were taken as 100%.

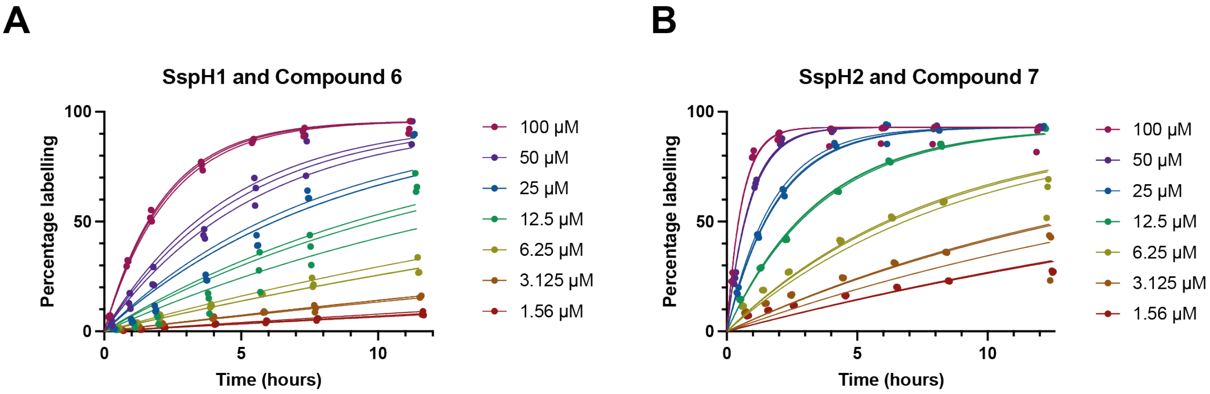

C

k_{inact}/K_I values ($M^{-1}s^{-1}$) (2 d.p.)

	**6**	**7**
SspH1	1.23 ± 0.02	1.38 ± 0.03
SspH2	6.91 ± 0.08	6.20 ± 0.05

Supplementary Figure 4

Full kinetics characterisation with SspH1 and SspH2 with compounds **6** and **7**. Time courses (0-12 hours) of compound labelling (100 – 1.56 μM) of SspH1 and SspH2 (0.5 μM) for A) SspH1 and compound **6**, and B) SspH2 and compound **7**. Measurements were performed in technical triplicates (shown on graphs). Labelling percentages were plotted against time in GraphPad Prism v.10, and curves fitted separately for each replicate using one-phase association, with constraints Y0 = 0 and plateau = highest labelling percentage. Graphs for SspH1 and compound **7**, and SspH2 and compound **6** are shown in Figure 3E and F. C) Slope values from graphs in Figure 3G and H were converted from μM^{-1} $hour^{-1}$ to $M^{-1}s^{-1}$ to give k_{inact}/K_I values. For SspH2 kinetics, 100 μM k_{obs} were not used to calculate k_{inact}/K_I, as they were outside the linear range. Reported errors are Standard Error, as calculated in GraphPad Prism v.10.

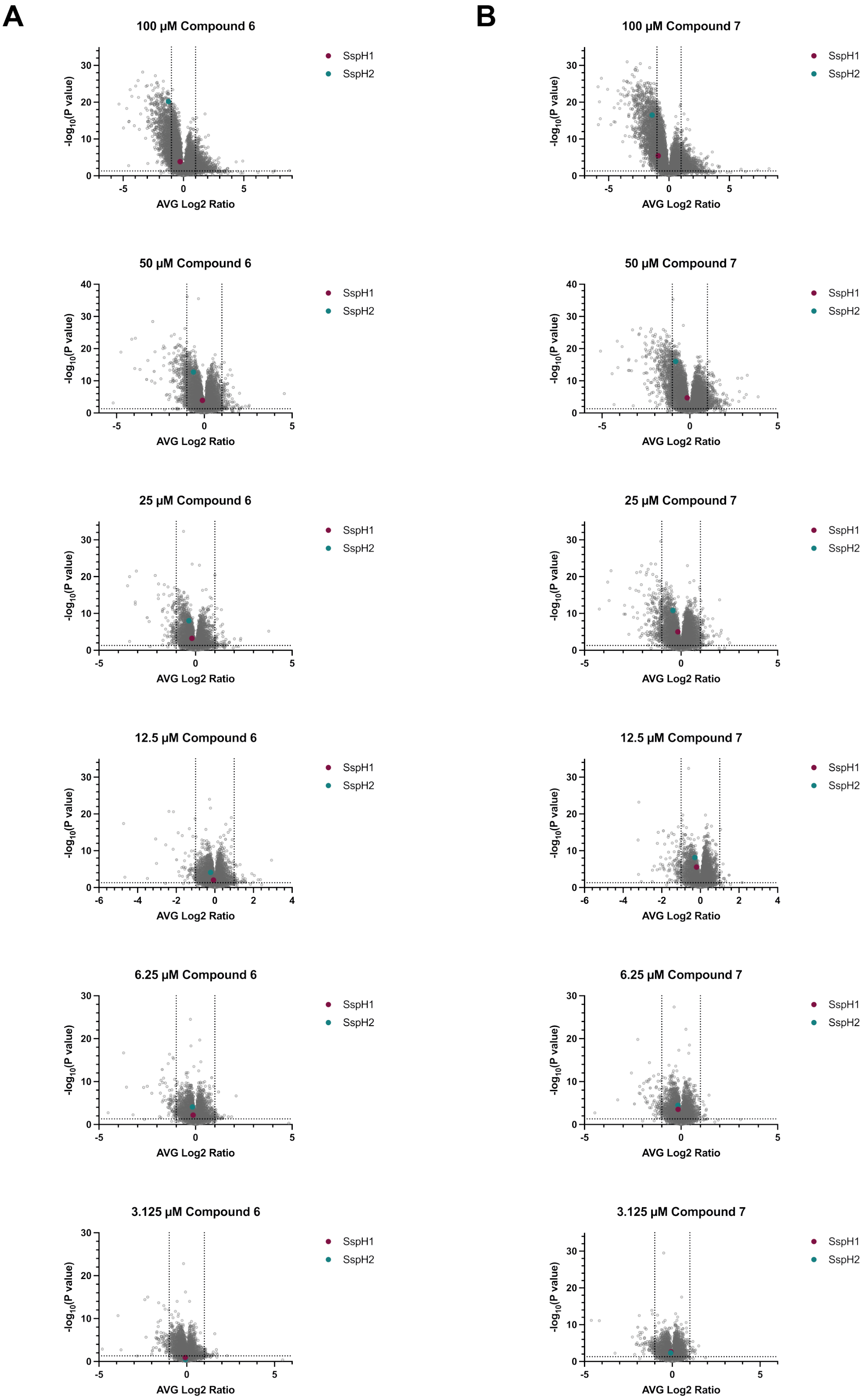

Supplementary Figure 5

Lysate proteomics volcano plots for A) compound **6** (100 – 3.125 µM) and B) compound **7** (100 – 3.125 µM) with all human peptides shown in grey. SspH1 and SspH2 peptides are highlighted in turquoise and purple. All proteomics experiments were performed with technical quadruplicates.

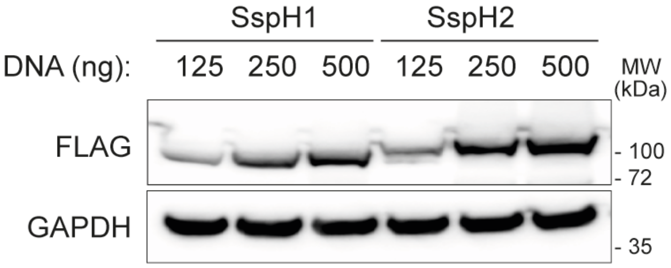

Supplementary Figure 6

Western blot of overexpressed FLAG-SspH1 and FLAG SspH2 in HEK293T cells with different quantities of DNA transfected.

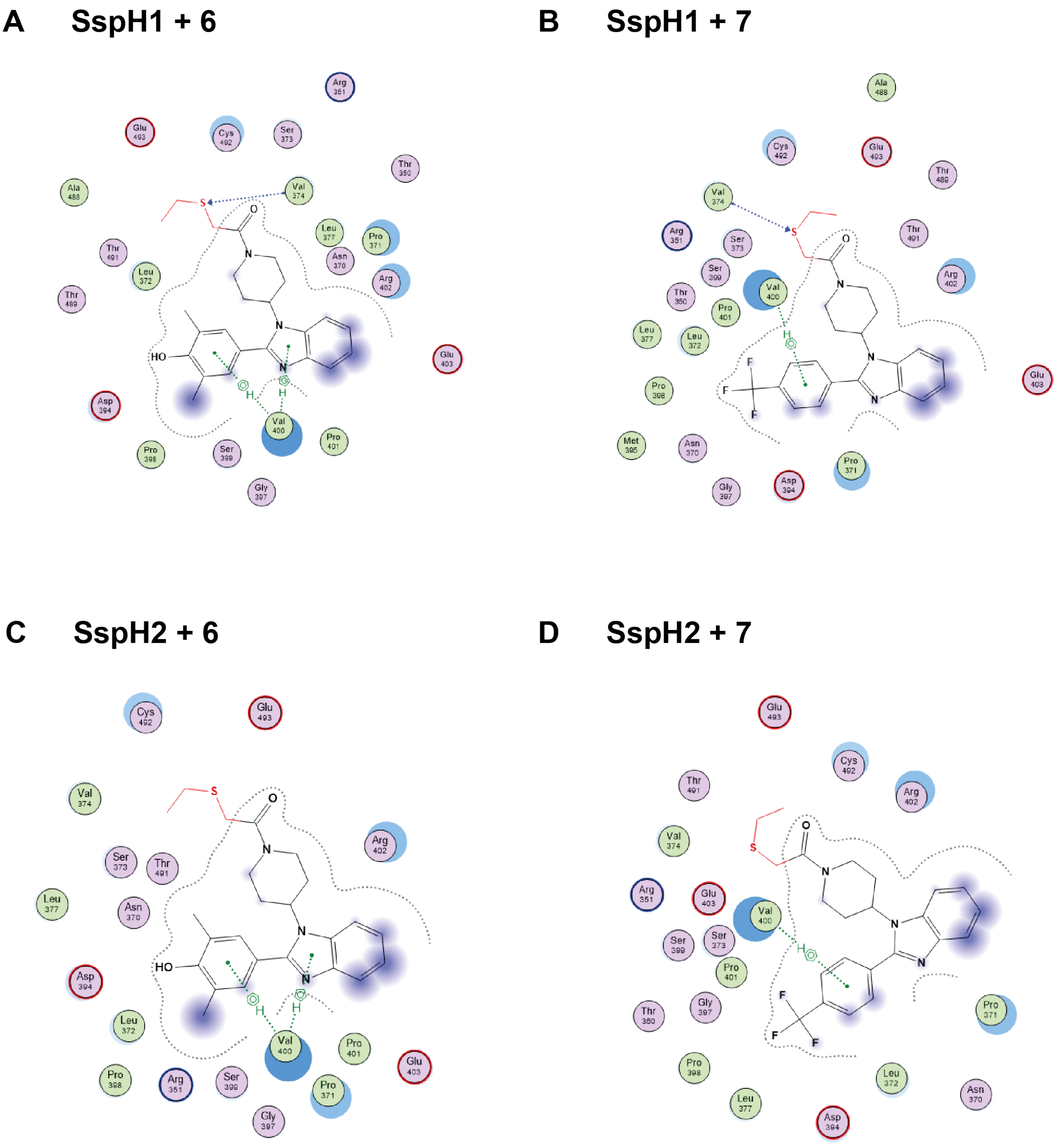

Supplementary Figure 7

Interaction maps for docking of A) SspH1 (PDB 9H6W) with compound **6**; B) SspH1 (PDB 9H6W) with compound **7**; C) SspH2 (PDB 3G06) with compound **6**; D) SspH2 (PDB 3G06) with compound **7**.

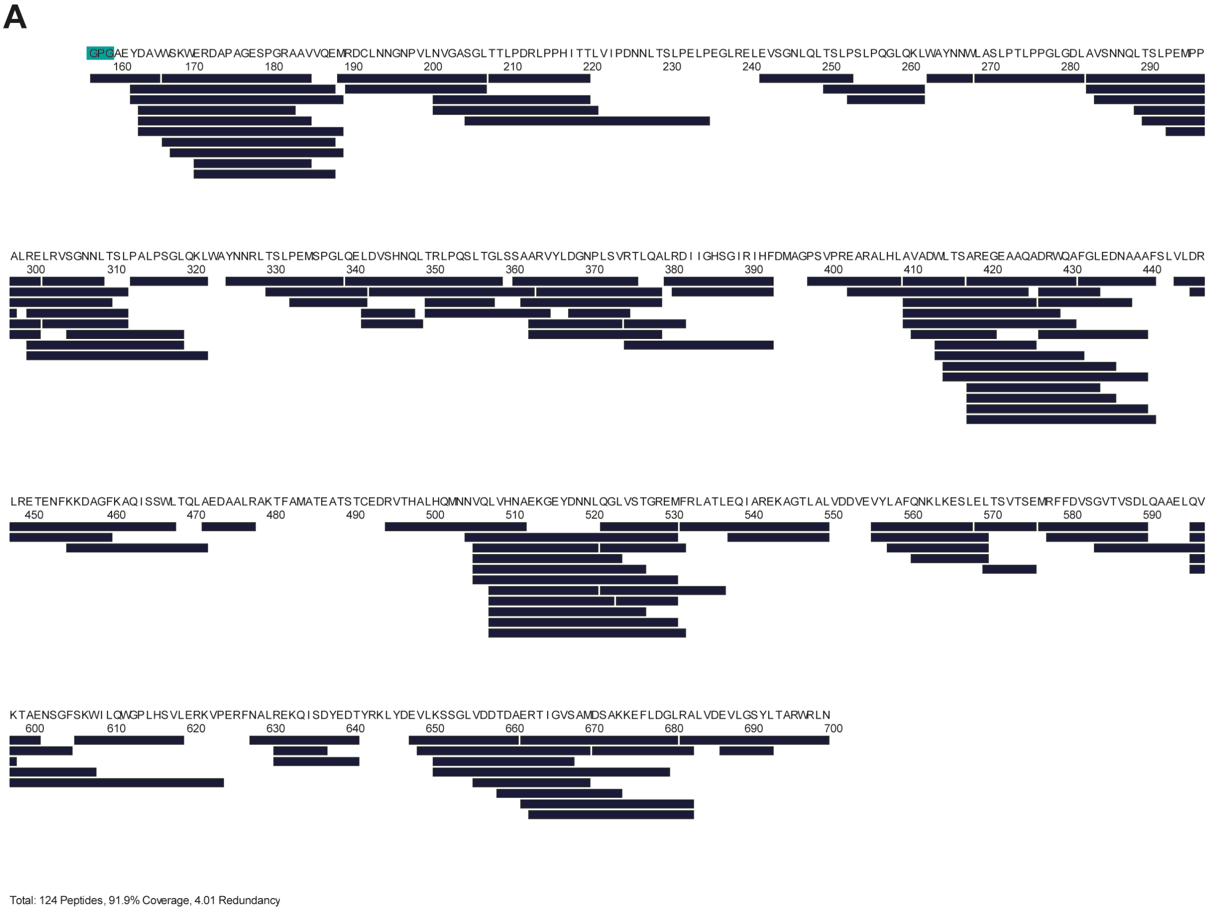

Supplementary Figure 8

HDX-MS data. SspH1 sequence coverage.

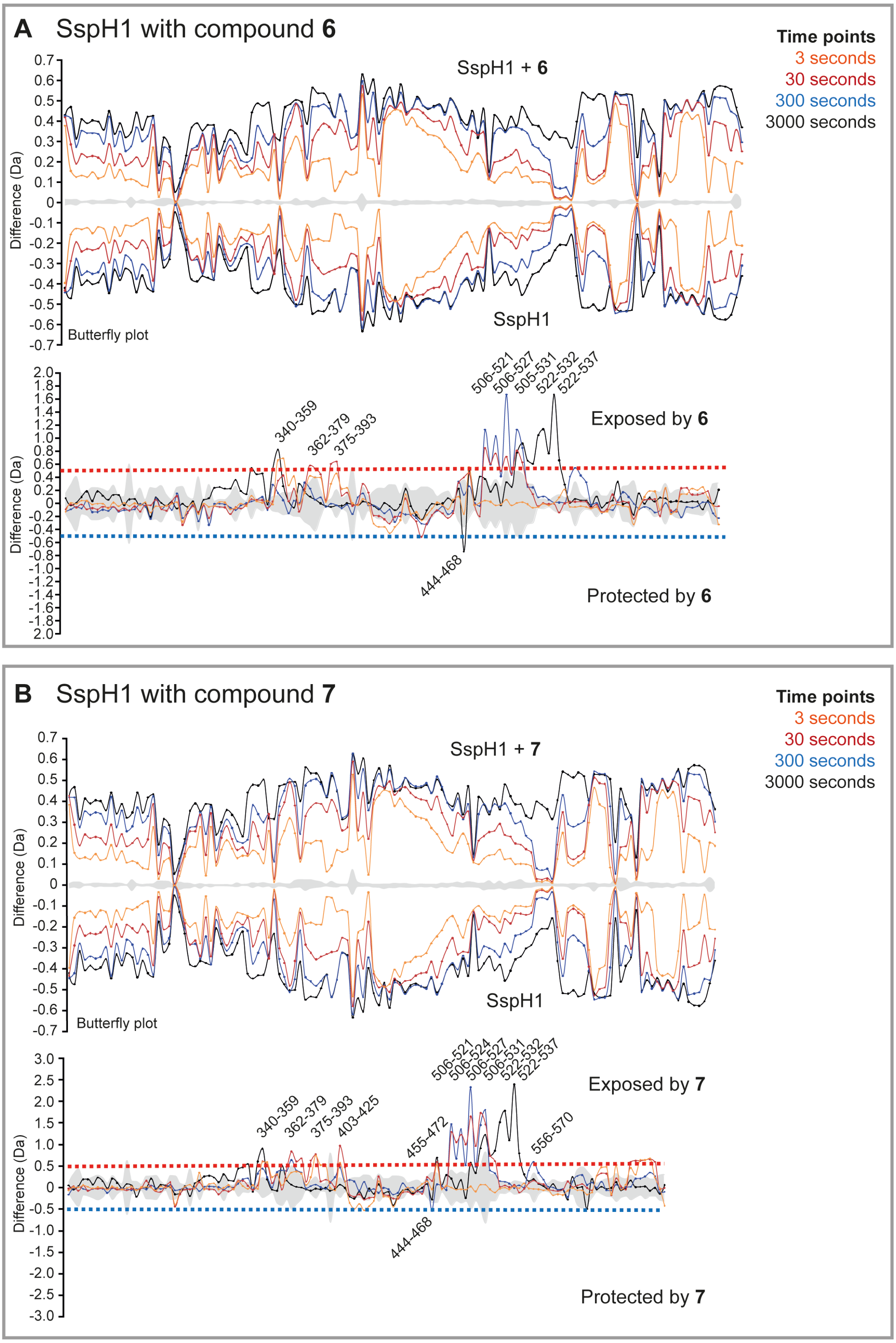

Supplementary Figure 9

HDX-MS data. Butterfly plots and exposed/protected peptides for SspH1 pre-treated with A) compound **6** and B) compound **7**.

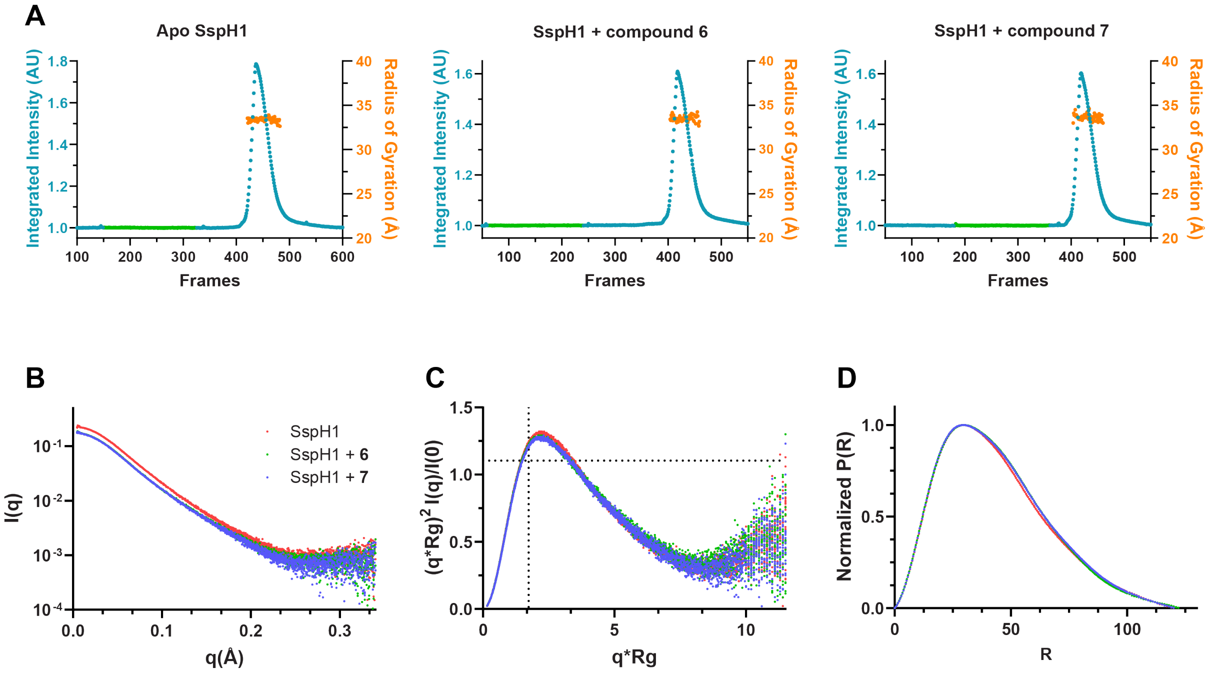

Supplementary Figure 10

SAXS data. A) Elution profiles and radii of gyration for apo SspH1 and SspH1 pre-treated with compound **6** or **7**. Frames used for buffer baseline subtraction are highlighted in green. B) X-ray scattering profiles, C) Dimensionless Kratky plot and D) normalized pair-distribution functions P(R) for apo SspH1 (red), SspH1 + **6** (green), and SspH1 + **7** (blue).

Supplementary Table 3: SAXS parameters.

**Data collection**			
Beamline	B21 at Diamond		
Wavelength	0.9464 Å		
q range ($Å^{-1}$)	0.0045 – 0.34		
Detector	EigerX 4M (Dectris)		
Beamsize	< 75 µm		
Energy	13.1 keV		
Column	Superdex 200 3.2 x 300 (total volume = 2.4 ml)		
Flow rate (ml/min)	0.075		
Temperature (°C)	15		
**Samples details**	**ΔSspH1**	**ΔNSspH1/compound 6**	**ΔNSspH1/compound 7**
Sample volume (µl)	40	40	40
Sample concentration (mg/ml)	10	10	10
**Structural parameters**			
**Reciprocal Space**			
Rg (Å) Guinier	34.4	34.4	34.6
I(0) (cm^{-1})	0.23	0.18	0.18
qRg limit	1.26	1.24	1.25
**Real Space**			
Rg (Å) P(R)	34.5 ± 0.02	34.5 ± 0.02	34.6 ± 0.03
I(0) (cm^{-1})	0.23000 ± 0.00007	0.18000 ± 0.00008	0.18000 ± 0.00007
Rc (Å)	19.1	19.2	19.3
Dmax (Å)	120	122	120
Porod volume ($Å^3$)	85157	85201	84938
**Molecular mass determination**			
Theoretical MW (kDa)	59.5	60.2	60.2
DATPOROD MW (kDa) (Vp/1.6)	53.2	53.2	53.1
SAXS MoW2 (q = 0.3 $Å^{-1}$)	61.5	60.8	62.7
